# The BNB–GLID module regulates germline fate determination in *Marchantia polymorpha*

**DOI:** 10.1093/plcell/koae206

**Published:** 2024-07-23

**Authors:** Xiaolong Ren, Xiaoxia Zhang, Xiaotong Qi, Tian Zhang, Huijie Wang, David Twell, Yu Gong, Yuan Fu, Baichen Wang, Hongzhi Kong, Bo Xu

**Affiliations:** State Key Laboratory of Systematic and Evolutionary Botany, Institute of Botany, Chinese Academy of Sciences, Beijing 100093, China; University of Chinese Academy of Sciences, Beijing 100049, China; State Key Laboratory of Systematic and Evolutionary Botany, Institute of Botany, Chinese Academy of Sciences, Beijing 100093, China; China National Botanical Garden, Beijing 100093, China; State Key Laboratory of Systematic and Evolutionary Botany, Institute of Botany, Chinese Academy of Sciences, Beijing 100093, China; University of Chinese Academy of Sciences, Beijing 100049, China; State Key Laboratory of Systematic and Evolutionary Botany, Institute of Botany, Chinese Academy of Sciences, Beijing 100093, China; University of Chinese Academy of Sciences, Beijing 100049, China; State Key Laboratory of Systematic and Evolutionary Botany, Institute of Botany, Chinese Academy of Sciences, Beijing 100093, China; University of Chinese Academy of Sciences, Beijing 100049, China; Department of Genetics and Genome Biology, University of Leicester, Leicester LE1 7RH, UK; State Key Laboratory of Systematic and Evolutionary Botany, Institute of Botany, Chinese Academy of Sciences, Beijing 100093, China; University of Chinese Academy of Sciences, Beijing 100049, China; State Key Laboratory of Systematic and Evolutionary Botany, Institute of Botany, Chinese Academy of Sciences, Beijing 100093, China; University of Chinese Academy of Sciences, Beijing 100049, China; University of Chinese Academy of Sciences, Beijing 100049, China; China National Botanical Garden, Beijing 100093, China; Key Laboratory of Photobiology, Institute of Botany, Chinese Academy of Sciences, Beijing 100093, China; State Key Laboratory of Systematic and Evolutionary Botany, Institute of Botany, Chinese Academy of Sciences, Beijing 100093, China; University of Chinese Academy of Sciences, Beijing 100049, China; China National Botanical Garden, Beijing 100093, China; State Key Laboratory of Systematic and Evolutionary Botany, Institute of Botany, Chinese Academy of Sciences, Beijing 100093, China; China National Botanical Garden, Beijing 100093, China

## Abstract

Germline fate determination is a critical event in sexual reproduction. Unlike animals, plants specify the germline by reprogramming somatic cells at the late stages of their development. However, the genetic basis of germline fate determination and how it evolved during the land plant evolution are still poorly understood. Here, we report that the plant homeodomain finger protein GERMLINE IDENTITY DETERMINANT (GLID) is a key regulator of the germline specification in liverwort, *Marchantia polymorpha*. Loss of the Mp*GLID* function causes failure of germline initiation, leading to the absence of sperm and egg cells. Remarkably, the overexpression of Mp*GLID* in *M. polymorpha* induces the ectopic formation of cells with male germline cell features exclusively in male thalli. We further show that Mp*BONOBO* (*BNB*), with an evolutionarily conserved function, can induce the formation of male germ cell-like cells through the activation of Mp*GLID* by directly binding to its promoter. The Arabidopsis (*Arabidopsis thaliana*) MpGLID ortholog, MALE STERILITY1 (AtMS1), fails to replace the germline specification function of MpGLID in *M. polymorpha*, demonstrating that a derived function of MpGLID orthologs has been restricted to tapetum development in flowering plants. Collectively, our findings suggest the presence of the BNB–GLID module in complex ancestral land plants that has been retained in bryophytes, but rewired in flowering plants for male germline fate determination.

## Teaser

The genetic module underlying germline fate determination has been rewired during the land plant evolution.

## Introduction

The life cycles of most eukaryotic organisms consist of 2 generations, namely, a haploid phase and a diploid phase. The alternation of 2 generations is a central basis for the evolution of living organisms (Bowman et al. 2016). Germ cells are a unique cell lineage in animals and plants that are committed to developing into haploid gametes (sperm and egg cells), which fuse to form a zygote to initiate the diploid phase (Walbot and Evans 2003). The establishment of the germ cell identity is a fundamental decision for living organisms to either remain somatic or to produce gametes for the completion of the life cycle. In animals, germline cells are specified from pluripotent stem cells at the early stage of embryogenesis (Zhao and Garbers 2002; Lesch and Page 2012; Tang et al. 2016). By contrast, the germline fate of plants is acquired in gametophytes via de novo reprogramming of somatic cells at a late developmental stage (Berger and Twell 2011; Schmidt et al. 2015). In flowering plants, the male germline is defined once generative cells are formed by the asymmetric division of microspores (Berger and Twell 2011; Huang et al. 2021). Due to highly reduced gametophytes, the establishment of male germline fate in flowering plants is coupled with microsporogenesis deep inside anthers, hindering the genetic exploration of this landmark event (Berger and Twell 2011; Nakajima 2018).

Bryophytes are resolved as the sister group to vascular plants and diverged from common ancestors of land plants around 450 million yr ago (Morris et al. 2018; One Thousand Plant Transcriptomes Initiative 2019; Naramoto et al. 2021). Unlike flowering plants, bryophytes possess a gametophyte-dominant generation. As a result, germline fate establishment and sporogenesis are separated temporally and spatially in bryophytes (Durand 1908; Ishizaki et al. 2016; Hackenberg and Twell 2019; Kohchi et al. 2021). When exposed to environmental stimuli, elaborate gametophytes switch from vegetative to reproductive growth and produce specialized sexual organs (male antheridia and female archegonia), where germline cells are specified and develop to form sperm and egg cells (Hisanaga et al. 2019; Kohchi et al. 2021; Bowman et al. 2022). The male germline initiates during the early stage of antheridium development. In the model bryophyte species, *Marchantia polymorpha*, an epidermal cell-derived antheridial initial cell (AIC) undergoes consecutive divisions forming an antheridial structure comprising 2 or 3 cell layers. The subsequent longitudinal division of these cells gives rise to inner cells, known as spermatogenous cells (SCs), which are square in shape with large nuclei and dense cytoplasm, indicating that the male germline fate is determined (Durand 1908; Zinsmeister and Carothers 1974; Shimamura 2016).

Recent advances have shed light on the germ cell specification of bryophytes (Hisanaga et al. 2019). In *M. polymorpha*, the basic helix–loop–helix (bHLH) transcription factor (TF) gene *BONOBO* (Mp*BNB*) was identified as a key regulator presumably mediating the germline fate entry (Yamaoka et al. 2018). Mp*BNB* is specifically expressed in gametangium initial cells and in sperm and egg progenitors, and the activation of Mp*BNB* induces reproductive development in vegetative growth conditions. Furthermore, in the moss *Physcomitrium patens*, 2 genes homologous to *Arabidopsis thaliana MALE STERILITY 1* (*AtMS1*), *PpMS1A* and *PpMS1B*, are also expressed in sperm and egg progenitors and required for gametogenesis in *P. patens* (Landberg et al. 2022). However, the genetic mechanisms operating in male germline fate determination remain relatively unknown. To understand the genetic changes underlying the evolution of the male germline establishment in land plants, we explored this remarkable event in *M. polymorpha*.

## Results

### Identification of genes specifically expressed at the early stage of antheridium development in *M. polymorpha*

**Figure 1.**
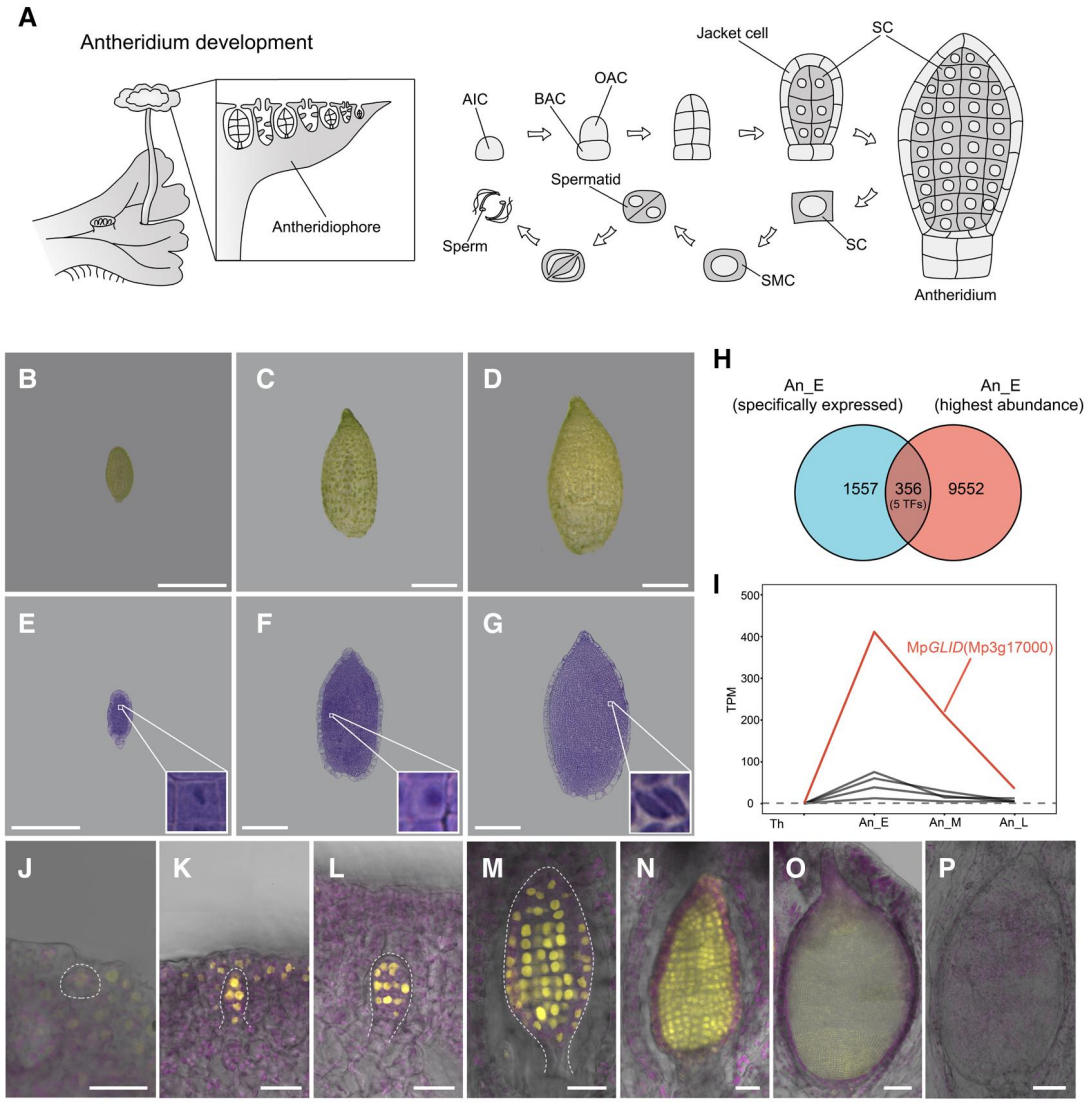
Identification of genes highly expressed in the early stage of antheridium development. **A)** Diagrams of the sperm cell formation in the antheridium from *M. polymorpha*. AIC, antheridial initial cell; BAC, basal antheridial cell; OAC, outer antheridial cell; SC, spermatogenous cell; SMC, spermatid mother cell. **B to G)** Antheridia at early **B, E)**, middle **C, F)**, and late **D, G)** developmental stages collected for the transcriptome analysis. Transverse sections of antheridia **E to G)** are corresponding to those in **B to D)**. Insets in **E to G)** are close-up views of the SC **E)**, SMC **F)**, and differentiating sperm cells **G)**. Scale bars, 200 *μ*m. **H)** Venn diagrams of genes specifically expressed and highly expressed in the early stage of antheridium development (An_E). TFs, transcription factors. **I)** Expression of the 5 TF genes identified in **H)** in 21-d thalli (Th) and antheridia at early (An_E), middle (An_M), and late (An_L) stages. Each measurement represents the mean of 4 biological replicates. **J to P)** Venus-NLS accumulation during antheridium development in a representative line *pro*Mp*GLID:Venus-NLS*-12. Venus fluorescence indicates the activities of the Mp*GLID* promoter. The magenta signals are chlorophyll autofluorescence. The dashed lines indicate AIC in **J)** and developing antheridia in **K to M)**. Scale bars, 20 *μ*m **J to N)**; 200 *μ*m **O, P)**.

*M. polymorpha* is a dioicous plant species. When exposed to far-red light, the apical meristem of thalli develops into modified branches, called antheridiophores and archegoniophores, where antheridia and archegonia, respectively, initiate and develop (Fig. 1A) (Durand 1908; Zinsmeister and Carothers 1974; Shimamura 2016). Morphological changes that occur during male gametogenesis are clearly defined in developing antheridia, which can easily be manipulated in *M. polymorpha* (Fig. 1A). Male gametogenesis initiates when SCs are specified. The SCs continue proliferating in a nearly synchronous manner before they develop into spermatid mother cells (SMCs). Following diagonal division, each SMC produces 2 triangular spermatids that eventually differentiate into biflagellate sperm cells.

Depending on the key events that occur during male gametogenesis, we investigated the global gene expression profiles of antheridia at 3 developmental stages via RNA sequencing (RNA-seq). We compared the RNA-seq profiles from the early stage (SC specification), middle stage (SMC formation), and late stage (sperm cell morphogenesis) (Fig. 1, B to G), with the RNA-seq data from 21-d-old thalli bearing gemma cups with gemmae, which are specialized for vegetative propagation (Supplementary Data Set 1). If the transcripts per million (TPM) value is greater than 1, we considered the gene to express in the sampled tissues. We identified 1,913 genes as early-stage antheridium-specific genes, of which 356 genes had peak expression at the early stage and decreased at the middle and late stages (Fig. 1H; Supplementary Data Sets 2 and 3). Among these 356 genes, 5 were annotated as TFs, including the bHLH TF gene *BONOBO* (Mp*BNB*), which is expressed in sperm cell progenitors in *M. polymorpha* (Supplementary Data Set 4; Yamaoka et al. 2018; Cui et al. 2023) and a Brassinazole-resistant 1/BRI1-EMS-Suppressor 1 (BZR/BES) TF gene Mp*BZR3*, which is essential for the early development of antheridium and late archegonium development (Furuya et al. 2024).

We focused on the gene, Mp3g17000, which encodes a plant homeodomain (PHD) finger protein (Fig. 1I) with a TPM value of approximately 5.4-fold greater than that of the second highest expressed gene in early-stage antheridia (Fig. 1I; Supplementary Data Set 4). This gene was also identified as an antheridium-specific TF by Higo et al. (2016), which was further supported by our reverse transcription quantitative PCR (RT-qPCR) analysis of 9-d-old thalli, 30-d-old thalli, gemmae, antheridiophores, and archegoniophores 28 d after reproductive induction (Supplementary Fig. S1). With these data, Mp3g17000 would be expected to be potentially associated with SC formation in *M. polymorpha*; therefore, we named this gene *GERMLINE IDENTITY DETERMINANT* (Mp*GLID*).

### Preferential expression of Mp*GLID* in developing antheridia

To understand the function of Mp*GLID* in *M. polymorpha*, we checked its temporal expression during antheridium development. We produced male transgenic *M. polymorpha* plants expressing the yellow fluorescent protein Venus fused with a nuclear localization signal (NLS) driven by a 4.8-kb-long Mp*GLID* promoter (*pro*Mp*GLID:Venus*-*NLS*) (Supplementary Fig. S2A). Although weak fluorescence was detected in the dorsal epidermis and filamentous photosynthetic cells of antheridiophores, the Mp*GLID* promoter was mainly active throughout the antheridium development (Fig. 1, J to O), as previously indicated by RNA in situ hybridization (Higo et al. 2016). The Venus-NLS protein accumulated in AICs (Fig. 1J), and we observed a strong fluorescence in both SCs and in the single-cell layer surrounding the SCs, termed jacket cells, in young antheridia (Fig. 1, K to M). During the antheridium development, the Venus signal was relatively restricted and preferentially detected in the inner cells of antheridium (Fig. 1N), and its intensity gradually diminished and became faint as sperm cell morphogenesis commenced (Fig. 1, O and P). These results suggest that MpGLID is preferentially expressed at the early stage of antheridium development and may play a role in male gametogenesis of *M. polymorpha*.

### Failure of SC specification in Mp*glid^ge^* mutants

To elucidate the MpGLID function in gametogenesis, we generated Mp*GLID* loss-of-function *M. polymorpha* mutants via clustered regularly interspaced short palindromic repeats (CRISPR)/CRISPR-associated protein 9 (Cas9)-mediated genome editing (ge) (Supplementary Fig. S2, B and C). Using a guide RNA targeted to the 5′-end of the Mp*GLID* coding region caused frame-shift mutations that introduced premature stop codons, leading to the complete loss of MpGLID. Given our focus on the formation of SCs, 2 independent lines of male Mp*glid^ge^* mutants were successfully established (Supplementary Fig. S3A).

Under experimental conditions, we observed no discernible changes in vegetative growth, induction of reproductive growth, or antheridiophore and archegoniophore development at a macroscopic level between Mp*glid^ge^* mutants and wild-type (WT) plants (Supplementary Fig. S3), with one exception, that is, the dorsal side of WT antheridiophores had brownish pigmentation, which we did not observe in Mp*glid^ge^* mutants (Supplementary Fig. S4, A and B). Given that the brownish pigmentation is associated with mature antheridia, the lack of pigmentation in Mp*glid^ge^* mutants implied aberrant antheridium development. Moreover, when we applied water to the top of the antheridiophores, the sperm cells were discharged from well-developed WT antheridiophores harboring mature antheridia, but no sperm cells were discharged from Mp*glid^ge^* antheridiophores at the same developmental stage (Supplementary Fig. S4, C and D). Together, these observations indicate that MpGLID is crucial for the antheridium development and has a key role in gametogenesis.

**Figure 2.**
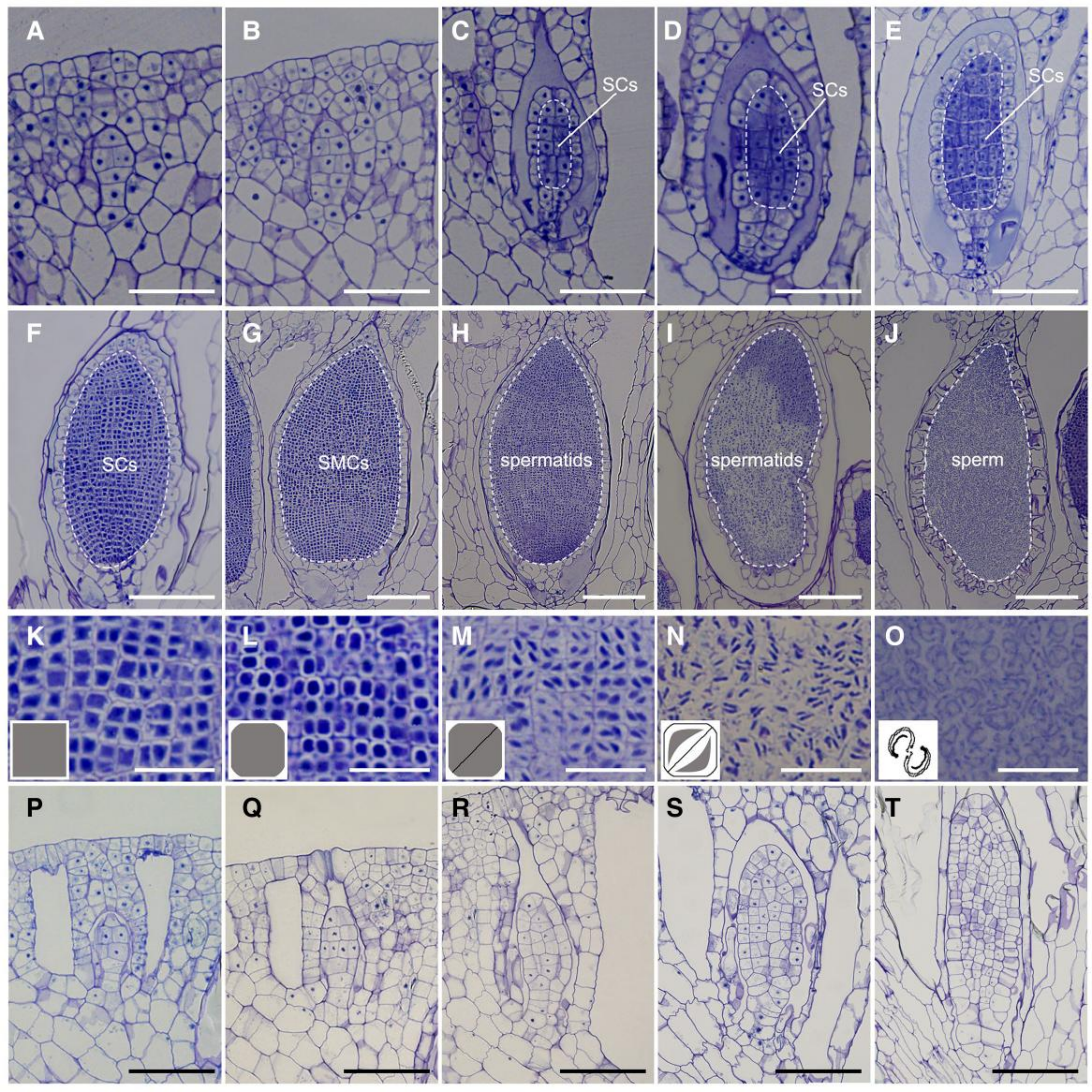
Mp*GLID* is essential for the SC specification in *M. polymorpha*. **A to J)** Antheridium development in the WT. The SCs are specified in **C)**. The white dashed lines indicate the inner cells of antheridia at various developmental stages. SCs, spermatogenous cells; SMCs, spermatid mother cells. Scale bars, 50 *μ*m **A to E)**; 100 *μ*m **F to J)**. **K to O)** Sperm cell differentiation in antheridia corresponding to the developmental stages in **F to J)**, respectively. Insets are schematic illustrations of sperm differentiation. Scale bars, 20 *μ*m. **P to T)** Antheridium development in a representative mutant line Mp*glid^ge^*-7. Note the absence of SCs in Mp*glid^ge^*-7. Scale bars, 50 *μ*m.

We further compared antheridium development in WT plants (Fig. 2, A to O) and Mp*glid^ge^* mutants (Fig. 2, P to T). In WT plants, SCs, the male germline cells in *M. polymorpha*, are characterized as a group of small, square-shaped cells each with a large nucleus and a dense cytoplasm (Fig. 2, C to F). By contrast, in Mp*glid^ge^* mutants, while the initiation of antheridium development was not affected (Fig. 2, A, B, P, and Q; Supplementary Fig. S5), these plants completely failed to specify typical SCs inside young antheridia (Fig. 2, R to T). Instead, these cells that were supposed to be SCs remained similar to their precursor cells, each with a less-compact nucleus and a sparse cytoplasm (Fig. 2, Q and R). These abnormal cells in Mp*glid^ge^* mutants underwent several rounds of irregular division, antheridium growth eventually aborted, and differentiation ceased at an early stage, leaving a group of unspecified inner cells without SC identity (Fig. 2, R to T). Jacket cell development was also affected in Mp*glid^ge^* mutants, as indicated by the failure of cell expansion and vacuolation (Fig. 2, R to T), suggesting that the progression of the jacket cell development requires a proper SC formation or MpGLID function in *M. polymorpha*.

### Ectopic induction of SC-like cells in male thalli by overexpression of Mp*GLID*

To better understand the MpGLID function in the male germline cell specification, we constitutively expressed Mp*GLID* under the control of the *M. polymorpha Elongation Factor 1 alpha* promoter in *M. polymorpha* (*pro*Mp*EF1α:*Mp*GLID*) (Supplementary Fig. S2D). Almost all transgenic plants exhibited a similar phenotype of retarded growth, more branches, and abnormal thalli. We randomly picked 7 independent transgenic lines to check the Mp*GLID* expression and selected 2 male lines and 2 female lines by high Mp*GLID* expression for further analysis (Supplementary Fig. S6A).

**Figure 3.**
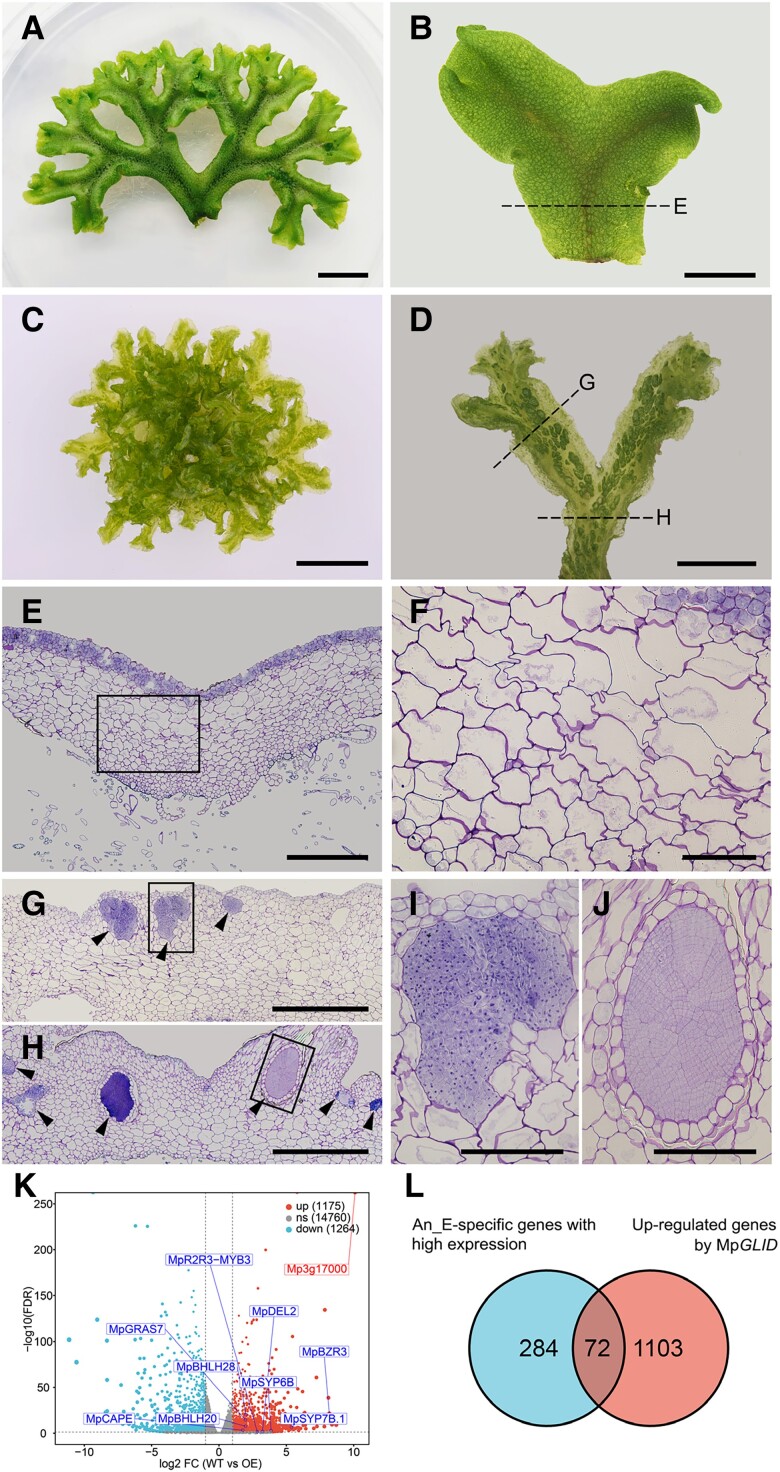
Mp*GLID* overexpression induces ectopic formation of SC-like cells in thalli. **A to D)** Dorsal views of a WT **A, B)** and a *pro*Mp*EF1α:*Mp*GLID*-18 plant **C, D)** 21 d after transplanting. Scale bars, 1 cm **A, C)** and 0.5 cm **B, D)**. **E)** Transverse section of thalli from WT indicated by the black dashed line in **B)**. Scale bar, 500 *μ*m. **F)** Close-up view of the area within the black box in **E)**. Scale bar, 100 *μ*m. **G, H)** Transverse sections of thalli from a representative overexpression line *pro*Mp*EF1α:*Mp*GLID*-18 indicated by the corresponding black dashed lines in **D)**. The arrowheads indicate the ectopic formation of SC-like cells. Scale bars, 500 *μ*m. **I, J)** Close-up views of SC-like cells within the black boxes in **G, H)**, respectively. Scale bars, 100 *μ*m. **K)** Volcano plot showing upregulated (log_2_FC > 1) and downregulated (log_2_FC< −1) genes induced by the Mp*GLID* overexpression in 21-d-old thalli. Genes reported to express in antheridium were upregulated and indicated on the volcano plot. Four biological replicates were used. **L)** Venn diagram of antheridium-specific genes expressed at the early development stage detected in Mp*GLID*-overexpressing thalli.

Although both *pro*Mp*EF1α:*Mp*GLID* and WT plants grew in a dichotomic pattern, the *proMpEF1α:MpGLID* lines had much smaller thalli (Supplementary Fig. S6, B to H). The *pro*Mp*EF1α:*Mp*GLID* plants developed rhizoids and scales on the ventral side of thalli, but the number of air chambers was substantially reduced on the dorsal side (Fig. 3, A to D; Supplementary Fig. S7, A to C). Furthermore, the *pro*Mp*EF1α:*Mp*GLID* plants did not develop gemma cups or gemmae (Fig. 3, A to D; Supplementary Fig. S7, A to C). Surprisingly, the histological staining of transverse sections of *pro*Mp*EF1α:*Mp*GLID* plants revealed clusters of cells with heavy toluidine blue staining in the parenchyma cell layer of male thalli (Fig. 3, E to J), but not in female thalli (Supplementary Fig. S7, D to I). These cells were distinct from their neighboring parenchyma and photosynthetic cells, but resembled SCs in some characteristics, such as their square shape, large nucleus, and dense cytoplasm. Although these SC-like cells were capable of proliferation, they were unable to develop into SMCs and therefore could not produce sperm. In some instances, the SC-like cells were encompassed by a well-recognized, single-layered jacket, forming a structure comparable to an antheridium (Fig. 3, H and J).

In line with the formation of SC-like cells, the RNA-seq analysis of 28-d-old male *M. polymorpha* thalli overexpressing Mp*GLID* revealed that numerous genes reported to predominantly express in developing antheridia, such as *COMBINED ADENYLYL CYCLASE* with *PHOSPHODIESTERASE* (Mp*CAPE*) (Kasahara et al. 2016; Yamamoto et al. 2024), *DP-E2F-LIKE2* (Mp*DEL2*) (Flores-Sandoval et al. 2018), and Mp*BZR3* (Flores-Sandoval et al. 2018; Furuya et al. 2024), were activated in these transgenic plants (Fig. 3K; Supplementary Data Set 5), which was further confirmed by the RT-qPCR analysis (Supplementary Fig. S8). Moreover, among the 356 genes previously identified as early antheridium-specific genes (Fig. 1H), 72 were dramatically upregulated in *pro*Mp*EF1α:*Mp*GLID* thalli (Fig. 3L; Supplementary Data Set 6). In summary, these findings indicate that the Mp*GLID* overexpression induces a transdifferentiation of vegetative cells to SC-like cells in thalli by activating an expression profile similar that in SCs; thus, MpGLID is a master regulator of the male germline fate determination in *M. polymorpha*.

### Mp*GLID* operates as a direct target of MpBNB to govern SC formation

A study in *M. polymorpha* has revealed that MpBNB is a key switch to initiating the development of sexual reproductive branches, antheridiophores and archegoniophores (Yamaoka et al. 2018). The specific and stable expression of Mp*BNB* is secured by the *bHLH* gene, *LOTUS JAPONICUS ROOTHAIRLESS LIKE* (Mp*LRL*), and CYTOKININ-INDEPENDENT 1 (CKI1)-mediated signaling in progenitors of sperm and/or egg cells at the early stages of antheridium and archegonium development, respectively (Yamaoka et al. 2018; Cui et al. 2023; Saito et al. 2023; Bao et al. 2024); however, its function in these cells is still unclear. We investigated the MpBNB function by generating transgenic *M. polymorpha* plants constitutively overexpressing Mp*BNB* under the control of the Mp*EF1α* promoter (*pro*Mp*EF1α:*Mp*BNB*).

**Figure 4.**
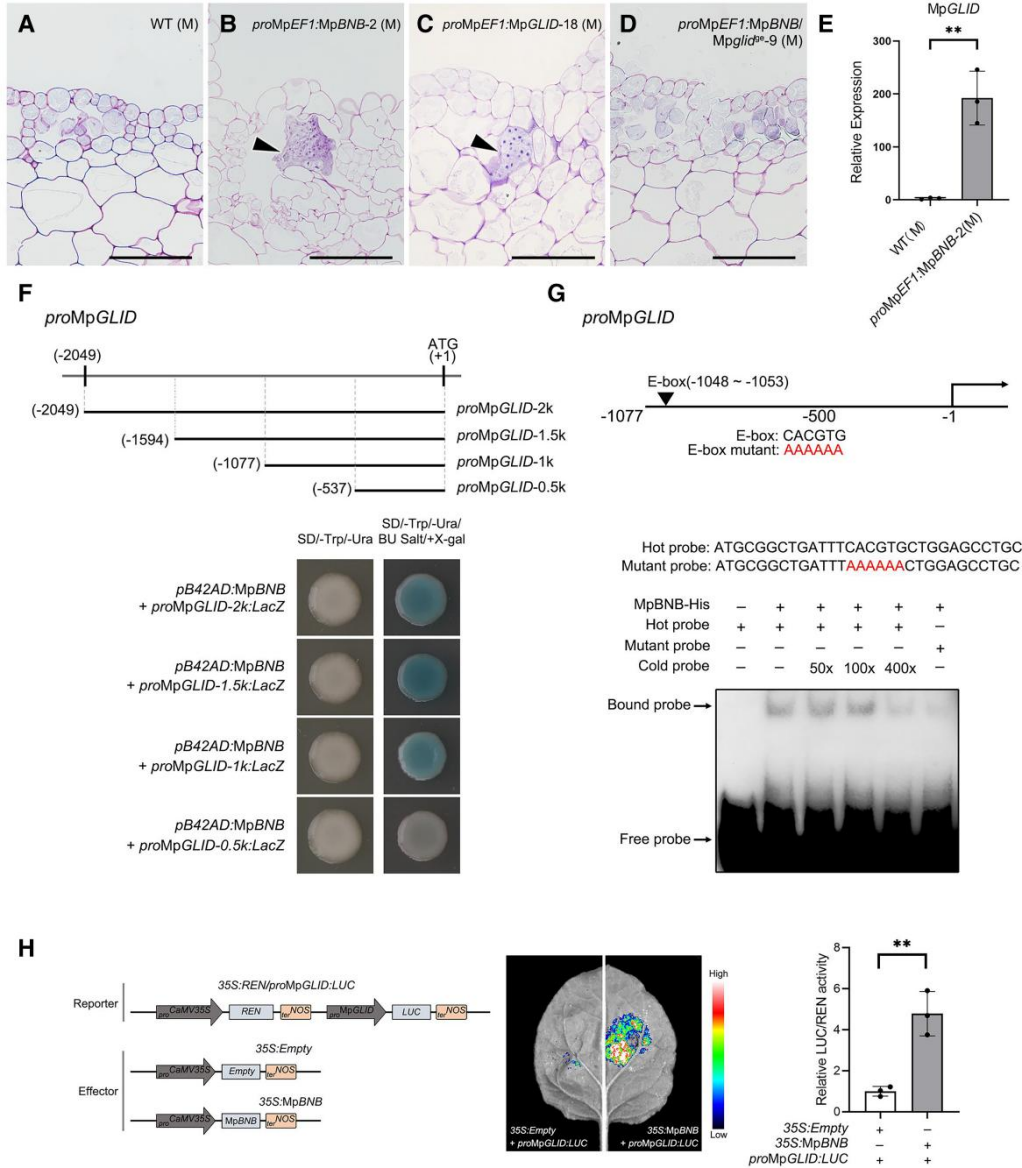
MpBNB induces a spermatogenous-like cell formation by the direct activation of the Mp*GLID* expression. **A to D)** Mp*GLID* is required for the MpBNB-mediated ectopic induction of SC-like cells in 21-d-old male thalli of *M. polymorpha*. Longitudinal sections of male thalli from WT **A)**, *pro*Mp*EF1α:*Mp*BNB***B)**, *pro*Mp*EF1α:*Mp*GLID***C)**, and *pro*Mp*EF1α:*Mp*BNB* Mp*glid^ge^*-9 **D)**. M, male. Scale bars, 100 *μ*m. **E)** RT-qPCR analysis. Dramatic upregulation of Mp*GLID* in thalli of *pro*Mp*EF1α:*Mp*BNB* plants. Each measurement represents the mean ± Sd of 3 biological replicates. **P < 0.01 (Student's *t* test). **F)** Yeast 1-hybrid assay. The deletion analysis of the Mp*GLID* promoter indicates that a region (from −1,077 to −537 bp) is responsible for the interaction with MpBNB. Yeast cells were grown on SD/-Trp/-Ura medium with BU Salt plus X-gal. **G)** EMSA assay. An E-box variant contributes to direct binding with MpBNB. The biotin-labeled WT (HOT) and mutated (Mutant) probes are indicated. Competition analysis was conducted with excess unlabeled WT probe (Cold) at 50×, 100×, and 400× amount of HOT probes. **H)** Luciferase reporter assay. MpBNB is able to activate transcription of the Mp*GLID* promoter in *N. benthamiana* leaves. Bioluminescence signals were imaged and quantified with the GloMax 20/20 Luminometer System to indicate the luciferase activities. Each measurement represents the mean ± Sd of 3 biological replicates. **P < 0.01 (Student's *t* test).

We confirmed the finding of Yamaoka *et al.* (2018) that overexpression of Mp*BNB* was able to induce a continuous formation of sexual branches under vegetative growth condition (Supplementary Fig. S9). Unexpectedly, clusters of cells with intensive staining of toluidine blue were detected in the parenchyma cell layer only of male transgenic thalli (Fig. 4, A and B). These cells shared cytological identities with SC-like cells induced by the overexpression of Mp*GLID*, including a dense cytoplasm and a large nucleus (Fig. 4C). Therefore, the expression of Mp*GLID* in *pro*Mp*EF1α:*Mp*BNB* thalli was evaluated.

It was found that the expression of Mp*GLID* significantly increased in the male thalli overexpressing Mp*BNB* (Fig. 4E). These observations led us to speculate that Mp*GLID* would be necessary for MpBNB to specify germline cells in *M. polymorpha*. Since the interruption of Mp*BNB* results in the failure of gametangiophore initiation and gametangia formation in *M. polymorpha* (Yamaoka et al. 2018), in order to test our speculation, we constitutively overexpressed Mp*BNB* in Mp*glid^ge^* mutants (Supplementary Fig. S10) and checked whether SC-like cells would be ectopically induced in male transgenic thalli. As expected, no SC-like cells were detected (Fig. 4D). Collectively, these results indicate that MpBNB functions upstream of MpGLID to dictate germline fate acquisition.

We next asked whether MpBNB directly controls the expression of Mp*GLID*. Yeast 1-hybrid assays were performed to explore the direct DNA–protein interaction between the Mp*GLID* promoter and MpBNB. MpBNB fused with GAL4 transcriptional activation domain activated expression of the reporter gene *LacZ* driven by a 2.0-kb promoter sequence of Mp*GLID* (Fig. 4F; Supplementary Fig. S11). Further analysis with the deleted Mp*GLID* promoter sequences showed that a fragment from −1,077 to −537 bp is responsible for the interaction with MpBNB (Fig. 4F). The TFs of the bHLH family can recognize and bind to similar DNA motifs (CANNTG), known as an E-box (Gu et al. 2014; de Martin et al. 2021; Michael et al. 2023). Within this fragment, we identified an E-box variant (CACGTG).

To validate whether this E-box variant motif contributes to the direct interaction, we carried out electrophoretic mobility shift assays (EMSA). MpBNB fused with His-tag (MpBNB-His) was able to directly bind to the 30-bp E-box-harboring fragment (Fig. 4G), corresponding to −1,066 to −1,037 bp of the MpGLID promoter sequence. However, the mutation of this E-box motif greatly reduced such interaction. Consistent with these findings above, the luciferase reporter assay using *Nicotiana benthamiana* leaves also showed MpBNB-dependent transcriptional activation of the Mp*GLID* promoter (Fig. 4H). Taken together, these results demonstrate that MpBNB activates the transcription of Mp*GLID* by directly binding to its promoter.

### MpGLID is also indispensable for female germ cell development

**Figure 5.**
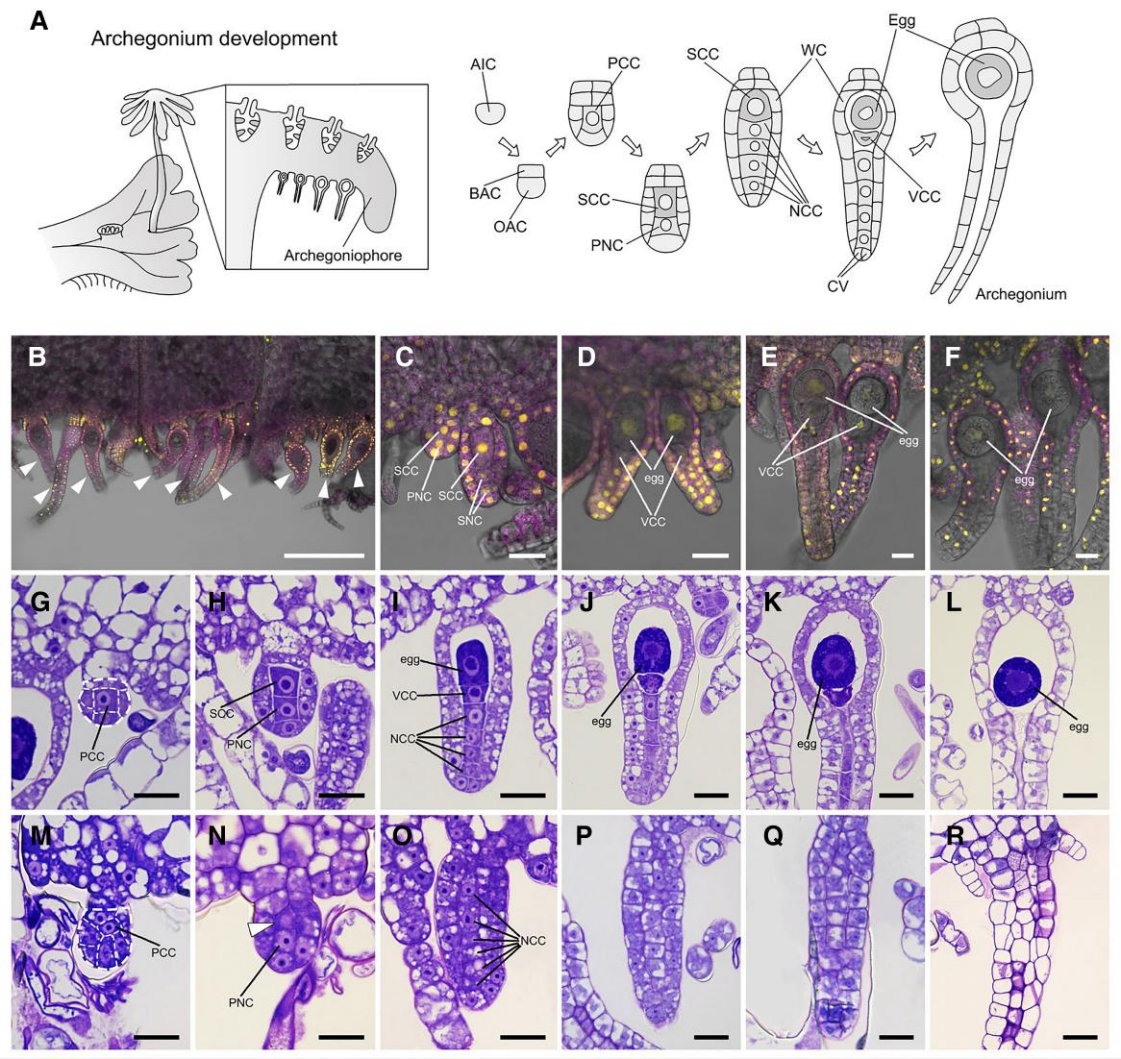
Mp*GLID* is critical for female germline formation in *M. polymorpha*. **A)** Diagrams of egg cell formation in the archegonium from *M. polymorpha*. AIC, archegonial initial cell; BAC, basal archegonial cell; OAC, outer archegonial cell; PCC, primary central cell; SCC, secondary central cell; PNC, primary neck canal; VCC, ventral canal cell; NCC, neck canal cell; CV, cover cell; WC, wall cell. **B to F)** Accumulation of Venus-NLS during archegonium development in a representative transgenic line *pro*Mp*GLID:Venus-NLS*-12. Strong Venus signals were observed in the SCC and faded away at the egg development initiation. Arrowheads indicate archegonia. Magenta signals are chlorophyll autofluorescence. Scale bars, 200 *μ*m **B)** and 20 *μ*m **C to F)**. **G to R)** Archegonium development in WT **G to L)** and Mp*glid^ge^*-8 **M to R)**. The cells comprising the young archegonium are indicated by dashed lines in **G)** and **M)**. The arrowhead in **N)** indicates the central cell without an SCC identity. Scale bars, 20 *μ*m.

Since male Mp*glid^ge^* mutants are sterile, we generated female Mp*glid^ge^* mutants and sought to cross them with male WT plants to produce transfer-DNA-free Mp*glid^ge^* mutants (Supplementary Fig. S3). However, we were unable to obtain sporophytes (Supplementary Fig. S12, A and B), suggesting that egg formation and/or archegonium development may be affected by the loss of Mp*GLID*. We therefore generated female *pro*Mp*GLID:Venus-NLS* plants to examine in detail whether Mp*GLID* is expressed during archegonium development. In *M. polymorpha*, an epidermal cell-derived archegonial initial cell (AIC) develops into a young archegonium with a primary central cell (PCC). This cell is segmented unequally to generate a secondary central cell (SCC) and a primary neck canal (PNC). The SCC enlarges and divides asymmetrically to form a ventral canal cell (VCC) and a large cell that ultimately matures into an egg cell (Fig. 5A). We detected the Mp*GLID* promoter activity in the entire archegonium throughout its development (Fig. 5, B to F) and observed a strong activity in the SCC that gives rise to the egg cell (Fig. 5C). Once the egg precursor cell is formed by SCC segmentation, it starts to develop into a mature egg cell. During this process, the Venus signal sharply decreased and eventually disappeared at egg cell maturity (Fig. 5, D to F).

Considering that Mp*GLID* is expressed in the archegonium, we observed the archegonium structure and development in female WT plants and Mp*glid^ge^* mutants (Supplementary Fig. S12, C to F). Unlike the flask-shaped structure in WT plants, the archegonia in Mp*glid^ge^* mutants were cylindrical and lacked the swollen venter of the WT (Supplementary Fig. S12, E and F). We further followed the archegonium development in Mp*glid^ge^* mutants (Fig. 5, G to R). In WT plants, the SCC with a conspicuous large nucleus and a dense cytoplasm enlarged and divided asymmetrically to produce 2 daughter cells, an egg cell and a VCC (Fig. 5, H and I). In Mp*glid^ge^* mutants, the development of young archegonia with a PCC was comparable to that of WT plants (Fig. 5, G and M). However, after PCC division, it failed to generate the SCC (Fig. 5N). The cell that was supposed to develop into SCC did not have an SCC morphology. Instead, this cell lost the compact nucleus and consequently failed to undergo enlargement and further cell divisions that normally produce the egg cell (Fig. 5, N and O). The other daughter cell derived from PCC had uncoordinated cell division, resulting in formation of unorganized cells situated in the venter and neck of the archegonium (Fig. 5, N to R). Collectively, these results demonstrate that MpGLID is also essential for specifying the SCC identity in female germline cell formation in *M. polymorpha*.

### Divergent function of MpGLID orthologs in flowering plants

MpGLID belongs to the ancient PHD family clade IIa (Cao et al. 2018; Landberg et al. 2022), which has split into 2 subclades (I and II) after the divergence of streptophyte lineages from chlorophyte algae, but before the streptophytes diversified (Supplementary Fig. 13A). Land plant proteins in subclade I further divided into 2 groups (groups A and B) probably due to a duplication event, which occurred in the common ancestor of land plants. Group A is flowering plant specific, while MpGLID is assigned to group B, together with its orthologs from nonseed land plants and gymnosperms, forming a sister clade to the orthologs of flowering plants.

Recent studies show the roles of group B proteins in flowering plants to be comparable (Ito et al. 2007; Yang et al. 2007; Li et al. 2011; Fernández Gómez and Wilson 2014; An et al. 2020; Han et al. 2023). In *A. thaliana*, AtMS1 is transiently expressed in tapetal cells, and AtMS1 controls the expression of genes associated with tapetal cell development as well as biosynthesis of pollen wall and coat materials, supporting the development of microspores into pollen (Ito and Shinozaki 2002; Vizcay-Barrena and Wilson 2006; Ito et al. 2007; Yang et al. 2007; Reimegård et al. 2017; Lu et al. 2020). Although the phylogenetic relationship between bryophytes and flowering plants is distant, we assessed whether AtMS1 of *A. thaliana* can complement the MpGLID function of *M. polymorpha*. Given the constitutive activity of the CRISPR/Cas9 cassette, a CRISPR/Cas9-resistant version of Mp*GLID* (Mp*GLID^re^*) was introduced. The expression of Mp*GLID^re^* driven by Mp*GLID* promoter (*pro*Mp*GLID:*Mp*GLID^re^*) restored the germline specification of male and female Mp*glid^ge^* mutants (Supplementary Fig. S13, B to G). By contrast, the expression of *AtMS1* under control of the Mp*GLID* promoter (*pro*Mp*GLID:AtMS1*) was unable to rescue the phenotype of germline specification defects in Mp*glid^ge^* mutants (Supplementary Fig. S13, H and I).

PHD finger domains are known for recognitions of specific histone modifications (Musselman and Kutateladze 2011; Sanchez and Zhou 2011; Mouriz et al. 2015). A typical PHD finger domain is the only recognized domain in PHD family clade IIa members (Supplementary Fig. S14). Specific histone marks and direct target genes bound by AtMS1 and its orthologs are still unclear *hitherto*. Considering that the overexpression of Mp*GLID* induced dramatic changes of thalli development (Supplementary Fig. S6, B to H), to better understand the functional difference between MpGLID and AtMS1, chimeric constructs were prepared to swap the PHD finger domains between MpGLID and AtMS1 (Supplementary Fig. 15A) and introduced into *M. polymorpha*. Overexpression of these 2 chimeric proteins failed to phenocopy the effects of MpGLID overexpression in *M. polymorpha* thalli (Supplementary Fig. 15B). Taken together, these findings indicate that molecular properties are not shared between MpGLID and AtMS1, and suggest that functional difference between these 2 proteins may possibly be due to discrete changes in both N-terminals and PHD finger domains.

The functions of genes from subclade II of PHD family clade IIa have not been reported to date. Mp3g06700 from this subclade was also identified as an antheridium-specific TF by Higo et al. (2016). However, this gene was not identified from the criteria (specific expression in antheridium and maximal abundance at the early stage of antheridium development), which we used to search for candidate genes most likely involved in the SC specification. The transcripts of Mp3g06700 gradually accumulated during antheridium development and reached maximum abundance only at late stage, which was 4-fold greater than that in early-stage antheridia (Supplementary Fig. 16A). Loss of the Mp3g06700 function did not influence male gametogenesis in mutants (Supplementary Fig. S16, B to D), indicating that this gene may not contribute to male gametogenesis. Interestingly, the ortholog AT1G33420 is almost ubiquitously expressed in *A. thaliana*, with a relatively higher transcript abundance in seeds and shoot apex (Supplementary Fig. S17). These observations suggest that subclade II proteins possibly may not play major roles in male gametogenesis in land plants.

## Discussion

Gamete formation is a prerequisite step for sexual reproduction. Adaptive changes of male gametogenesis have occurred in the plant evolutionary history to secure successful fertilization in terrestrial environments. As one of major lineages, bryophytes are considered to retain many ancestral traits of land plants (Rensing et al. 2020; Bowman et al. 2022). A comparative analysis of gene expression profiles in *M. polymorpha* using antheridia at various developmental stages with those of vegetative tissues revealed numerous genes specifically expressed at the key stages of antheridium development (Supplementary Data Sets 2 and 3). Most of these genes and their putative orthologs have not been functionally characterized in nonseed land plants or seed plants. Therefore, our data yield advanced genetic information associated with the core processes of male gametogenesis in bryophytes and provide a pool of potential genes to explore land plant lineage-specific diversification as well as the evolution of genetic pathways underlying male gametogenesis, particularly in flowering plants.

Considerable advances have been made in the understanding of male and female gametogenesis in flowering plants (McCormick 2004; Yadegari and Drews 2004; Borg et al. 2009; Pinto et al. 2019). A group of RWP-PR domain (RKD) TFs was implicated to mediate egg cell specification, and ectopic overexpression of some *RKD* genes in Arabidopsis was able to induce cell proliferation with an egg cell-specific transcription profile (Kőszegi et al. 2011; Koi et al. 2016; Rövekamp et al. 2016). Although key switches regulating the progression of female gametogenesis remain to be identified, comparative studies indicate substantial distinctions between genetic pathways driving male and female gametogenesis in flowering plants (Yang and Sundaresan 2000; Liu and Qu 2008; Hisanaga et al. 2019).

In this study, we showed that MpGLID functions as a master regulator in *M. polymorpha*, which is required not only for the SC specification in antheridia (Figs. 2 and 3), but also for the SSC formation in archegonia (Fig. 5). These results demonstrate that unlike flowering plants, a fundamental program controlled by GLID is shared between male and female gametogenesis in bryophytes, despite tremendous distinctions between their developmental processes. These data further suggest that the divergent genetic pathways responsible for male and female gametogenesis in flowering plants may result from the adaptive evolution of transient male and female gametophytes, which are dependent on sporophytes and embedded in 2 highly specialized sporophytic organs with distinct internal environments, anthers and ovaries, respectively.

A recent study in the moss *P. patens* revealed that *PpMS1A* and *PpMS1B* are required for gametogenesis in *P. patens* (Landberg et al. 2022). Cytological evidence supports a role for these 2 paralogous genes in the proliferation and differentiation of gamete-producing inner cells of gametangia, but molecular evidence for their functions in gametogenesis is still elusive. Here, we advanced the understanding of the functions of PHD proteins from group B of clade IIa by showing that MpGLID functions as a key regulator to switch on male and female germline fate in *M. polymorpha* (Figs. 2 and 5), which suggests a conserved role in bryophytes. Our results provide further evidence to indicate that MpBNB mediates the male germline fate entry, probably through a direct activation of the Mp*GLID* transcription (Fig. 4).

**Figure 6.**
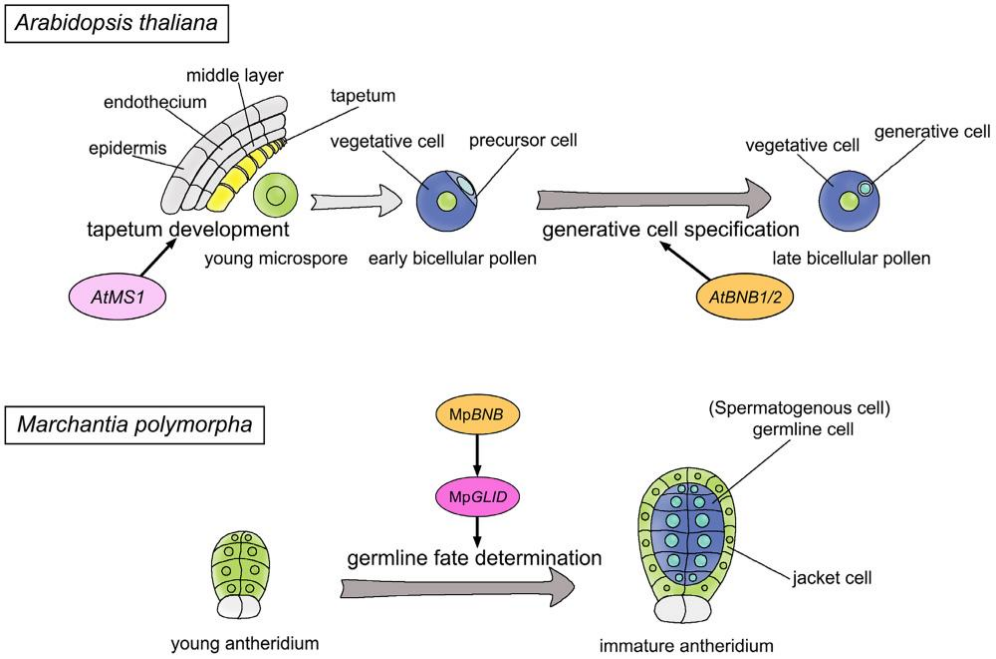
Evolution of the *BNB*–*GLID* module in land plants. Schematic illustration of the germline specification in *A. thaliana* and *M. polymorpha*. In *A. thaliana*, bHLH TFs (*AtBNB1/2*) control the generative cell specification, and the PHD-TF (*AtMS1*) exclusively regulates tapetum development. In *M. polymorpha*, the Mp*BNB*-Mp*GLID* module controls the SC formation. Orthologous genes are indicated in the same colors. A *BNB*–*GLID* module regulating male germline fate establishment may be present in the last ancestral land plants shared by bryophytes and vascular plants. This module has been preserved in bryophytes. During the flowering plant evolution, the genes orthologous to Mp*GLID* have been retained for their distinct role only in tapetum development of anthers, while *BNB* orthologs have a conserved role in the generative cell specification in pollen grains.

BNB proteins are members of the bHLH subfamily VIIIa, which is land plant specific (Pires and Dolan 2010; Bowman et al. 2017; Yamaoka et al. 2018). There are 2 BNB proteins in *P. patens*, and loss of PpBNB functions led to interruption of gametogenesis (Sanchez-Vera et al. 2022), suggesting the conserved function of BNB proteins in germline specification in land plants. Therefore, it is suggested that the function of the BNB–GLID module in determining male germline fate in the haploid gametophyte generation might be present in the last complex ancestral land plants, from which bryophytes and vascular plants were derived, and has been retained in bryophytes (Fig. 6).

As previously described about *PpMS1A* and *PpMS1B* in *P. patens* (Landberg et al. 2022), the Mp*GLID* promoter was also active in the diploid sporophyte generation, particularly in early developing embryos, foot cells of young sporophyte, and in developing sporangia (Supplementary Fig. S18). Although the function of Mp*GLID* and its *P. patens* orthologs in the sporophyte generation is still unknown, due to failure of sporophyte production by the corresponding mutants, similar patterns of spatio-temporal expression in *M. polymorpha* and *P. patens* suggest that these genes may potentially play roles in sporophyte development and/or sporogenesis in bryophytes, in addition to germline cell specification in the haploid gametophyte generation.

During the evolution of land plants, the haploid generation displays a trend of reduction in structural complexity (Bowman et al. 2016; Hackenberg and Twell 2019). Consequently, dramatic changes have occurred in male gametogenesis. In flowering plants, male gametogenesis is extremely reduced and confined to 3 cells produced by 2 rounds of mitosis in pollen grains (McCormick 1993; Borg et al. 2009). Accordingly, despite the conservation of the BNB function in the generative cell specification of flowering plants (Yamaoka et al. 2018; Sanchez-Vera et al. 2022), the function of the group B containing MpGLID in germline fate determination was lost in the haploid gametophyte generation. Instead, the function of genes in group B in the diploid sporophyte generation may have been restricted to tapetum development in flowering plants, which is critical for pollen formation (Fig. 6). Certainly, further investigation of MpBNB and MpGLID functions in sporophytes will be helpful to better understand the evolution of the BNB–GLID module in land plants.

## Materials and methods

### Plant materials and growth conditions

The bryophyte *M. polymorpha* strain “Cambridge” was used in this study (Xu et al. 2021). *M. polymorpha* was cultured on the half-strength of Gamborg's B5 media with vitamins containing 1% (w/v) agar at 22 °C under long-day conditions (16 h light:8 h dark) with LED light (120 *μ*mol/m^2^ s, ReLighting). To induce reproductive growth, 10-d-old plants cultured on agar plates were transplanted onto Jiffy-7 pellets (Jiffy Products International AS) in a growth room, the plants were exposed to far-red light (Philip) 30 d after transplantation to induce reproductive growth. *Nicotiana benthamiana* was grown at 22 °C under 16 h:8 h (light:dark) conditions in a growth room with LED light (150 *μ*mol/m^2^ s, ReLighting).

### RNA extraction and reverse transcription

The total RNA was extracted from isolated antheridia at early, middle, and late developmental stages and from 21-d-old thalli and from WT and *pro*MpEF1α:Mp*GLID* plants using the RNeasy Plant Mini Kit (Qiagen) following the manufacturer's instructions, and RQ1 RNase-Free DNase (Promega) was applied to remove the DNA contamination. The quality and quantity of the extracted total RNA were examined by a NanoDrop 2000 spectrophotometer (Thermo Fisher Scientific). Then, total RNA (1 *μ*g) was applied to synthesize the first-strand cDNA using SuperScriptII reverse transcriptase (Thermo Fisher Scientific) and oligo-(dT) primers. The reverse transcription product was used for further analysis.

### Quantitative RT-PCR

To examine the tissue-specific Mp*GLID* expression, 9-d-old thalli, 30-d-old thalli, gemmae, and antheridiophores and archegoniophores 28 d after reproductive induction were carefully harvested. To identify transgenic plants overexpressing Mp*GLID* or Mp*BNB*, thalli were collected 21 d after transplantation. The quantitative RT-PCR was conducted with a LightCycler 480 SYBR Green I Master kit (Roche) in a LightCycler 480 II (Roche). The oligonucleotide sequences are listed in Supplementary Data Set 7.

### Plasmid construction and plant transformation

To examine the Mp*GLID* expression in *M. polymorpha*, a binary plasmid pB34-KpnI-Venus-NLS harboring the yellow fluorescent protein Venus fused with nuclear localization signal peptide (*Venus-NLS*) was used (Delmans et al. 2017). An approximately 4.8-kb genomic fragment upstream of the Mp*GLID* start codon (*pro*Mp*GLID*) was amplified and inserted into the K*pn*I restriction enzyme site of the pB34-KpnI-Venus-NLS plasmid just before the *Venus-NLS* cassette by Gibson assembly (Gibson et al. 2009).

To ectopically overexpress Mp*GLID* and Mp*BNB* in *M. polymorpha*, a binary plasmid, pB34-PmeI-proMpEF1α, with a constitutive expression promoter, *pro*MpEF1α, was used (Delmans et al. 2017). The Mp*GLID* and Mp*BNB* coding regions were individually amplified and recombined into the P*me*I restriction enzyme site of the pB34-PmeI-proMpEF1α plasmid after *pro*MpEF1α by Gibson assembly (Gibson et al. 2009).

To mutate Mp*GLID*, Mp*BNB*, and Mp3g06700 in *M. polymorpha*, the 2-vector CRISPR/Cas9 system was applied as previously reported (Sugano et al. 2018). The plasmid pB45-proMpEF1α-hCas9 was used to constitutively express hCas9 under the control of *pro*Mp*EF1α*, and the other plasmid pB34-gRNA contained the *M. polymorpha U6* promoter (*pro*Mp*U6*) driving the transcription of the guide RNA. The CRISPR sequence targeting the 5′-end of the gene coding regions was designed by CasFinder (https://marchantia.info/tools/casfinder/). The synthesized CRISPR sequences with 15-bp overhangs identical to both flanking sequences of the insertion site right after *pro*Mp*U6* was recombined into pB34-gRNA by Gibson assembly (Gibson et al. 2009).

To constitutively overexpress Mp*BNB* in Mp*glid^ge^* mutants, the Mp*BNB* coding region was inserted into the P*me*I restriction enzyme site of the pB34-PmeI-proMpEF1α plasmid (Delmans et al. 2017). The resultant plasmid was used for the cotransformation of *M. polymorpha* sporelings with pB45-proMpEF1α-hCas9 and pB34-gRNA plasmids (Sugano et al. 2018) to generate the Mp*glid^ge^* mutants overexpressing Mp*BNB*.

To understand functional differences between MpGLID and AtMS1, their PHD finger domains were swapped, and chimeric sequences and the *AtMS1* coding sequence were separately introduced into pB34-PmeI-proMpEF1α (Delmans et al. 2017) by Gibson assembly (Gibson et al. 2009). For complementation analyses, the coding regions of Mp*GLID^re^* and *AtMS1* were amplified and separately introduced into pB34-PmeI under control of *pro*Mp*GLID* by Gibson assembly (Gibson et al. 2009).

All constructed plasmids described above were introduced separately into *Agrobacterium tumefaciens* strain GV3101 to generate the corresponding transgenic *M. polymorpha* plants, as previously reported (Ishizaki et al. 2008). The oligonucleotide sequences are listed in Supplementary Data Set 7.

### RNA-seq analysis

For the RNA-seq analysis, 4 biological replicates were tested. The total RNA samples were sent to BGI (Wuhan, China), and the quality was evaluated using the RNA 6000 Nano Kit (Agilent) on a Bioanalyzer 2100 (Agilent). Paired-end sequencing was performed on the BGI-DNBSEQ platform. Raw reads were processed by BGI, and over 10 Gb clean reads were provided. The *M. polymorpha* standard reference genome sequence and annotation MpTak_v6.1 were retrieved from MarpolBase (https://marchantia.info/download/MpTak_v6.1/). Read mapping was done against this genome sequence via HISAT2 (Kim et al. 2015), and quantification of gene expression was done via FeatureCounts (Liao et al. 2014). TPM were calculated. Raw counts were subjected to edgeR (Robinson et al. 2010) to identify differentially expressed genes.

### Microscopy observations

To examine the activity of the Mp*GLID* promoter, antheridiophores and archegoniophores 28 d after reproductive induction were collected from *pro*Mp*GLID:Venus-NLS* lines and fixed with 4% (w/v) paraformaldehyde in 100 mm sodium phosphate buffer (pH 7.0) at 4 °C for 1 h. The fixed antheridiophores were embedded in 5% (w/v) agar. Slices of 50 *μ*m thickness were produced using a VT1000 S vibratome (Leica). To investigate Mp*GLID* expression during sporophyte development, female *pro*Mp*GLID:Venus-NLS* lines were fertilized by sperm cells from male *pro*Mp*GLID:Venus-NLS* lines. Sporophytes were carefully harvested from various developmental stages. Images were captured using a Zeiss LSM 980 with Elyra7 confocal microscope (Zeiss). Samples were excited with 514 nm laser, and Detector Gain was 680 V. To observe antheridium and archegonium development, the antheridiophores and archegoniophores were fixed in 100 mm sodium phosphate buffer (pH 7.0) with 4% (w/v) paraformaldehyde overnight at 4 °C, and embedded in Technovit 7100 resin (Heraeus Kulzer) following the manufacturer's instructions. Semithin sections of 1 *μ*m were prepared using a RM2265 ultramicrotome (Leica). The sections were stained with 0.1% (w/v) toluidine blue O, and images were acquired with a DM6B microscope (Leica).

### Yeast 1-hybrid assay

The Mp*BNB* coding region was amplified and subcloned into the pB42AD vector (Lin et al. 2007) to generate the prey construct (pB42AD:Mp*BNB*). Mp*GLID* promoter sequences of different lengths were introduced into the pLacZ2u vector (Lin et al. 2007) to generate the bait constructs (*pro*Mp*GLID-2k:LacZ*, *pro*Mp*GLID-1.5k:LacZ*, *pro*Mp*GLID-1.0k:LacZ*, *pro*Mp*GLID-0.5k:LacZ*). These bait constructs were individually transformed into yeast (*Saccharomyces cerevisiae*) strain EGY48 (Clontech) together with the prey construct. Yeast cells were cultured on SD/-Trp/-Ura medium supplemented with X-gal (Thermo Fisher Scientific). Nuclear Factor Y B1 (NF-YB1) and the rice (*Oryza sativa*) *OsSUT4* promoter were used as the positive control (Bai et al. 2016). The oligonucleotide sequences are listed in Supplementary Data Set 7.

### EMSA

For expression of MpBNB-His recombinant protein, Mp*BNB* coding region was integrated into pET-28a plasmid. The resultant plasmid was introduced into *Escherichia coli* Rosetta-gami2 (DE3) strain (Biomed) and transformed *E. coli* cells were cultured in liquid LB media supplemented with 0.1 mm isopropyl β-D-1-thiogalactoside (IPTG, Thermo Fisher Scientific) at 22 °C overnight. The MpBNB-His recombinant protein was purified using a Mag-Beads His-Tag Protein Purification Kit (Sangon Biotech) following the manufacturer's instructions.

The labeled probes were prepared using an EMSA Probe Biotin Labeling Kit (Beyotime Biotechnology). EMSA assays were performed using a Chemiluminescent EMSA Kit (Beyotime) following the manufacturer's instructions. In brief, purified MpBNB-His protein and biotin-labeled probes were incubated at 22 °C for 20 min and electrophoresed using 6% (v/v) polyacrylamide gels. After that, biotin-labeled DNA was examined, and chemiluminescent signals were imaged with a Tanon 5200 Chemiluminescent Imaging System (Tanon). To validate the binding specificity, 50×, 100×, and 400× amount of unlabeled probe was added.

### Luciferase reporter assay

The promoter sequence of Mp*GLID* was subcloned into pGreenII-0080-LUC (Hellens et al. 2005) to generate the reporter plasmid *pro*Mp*GLID*:*LUC*. The coding region of Mp*BNB* was integrated into pGreenII-62-SK (Hellens et al. 2005) to generate the effector plasmid 35S:Mp*BNB*. The empty pGreenII-62-SK served as a negative control of the effector. These plasmids were individually transformed into *A. tumefaciens* strain GV3101 (pSoup) by electroporation. The transformed Agrobacterium cells were separately suspended in the infection solution (10 mm MES, 10 mm MgCl_2_, 0.2 mm acetosyringone, pH 5.6) and further mixed in a 1:1 volume ratio. The mixed suspensions were infiltrated into leaves of 6-wk-old *N. benthamiana*. Two days after infiltration, leaves were harvested and luciferase activities determined using the Dual-Luciferase Reporter Assay System (Promega) according to the manufacturer's instructions. Luminescence signals were imaged with the Tanon 5200 Chemiluminescent Imaging System (Tanon) and quantified with the GloMax 20/20 Luminometer System (Promega).

### Phylogenetic analysis

The genomes from 36 plant species across plant kingdom were obtained from Phytozome v.13 (https://phytozome-next.jgi.doe.gov/), PhycoCosm (https://phycocosm.jgi.doe.gov/phycocosm/home), and their public websites. The putative PHD proteins were retrieved by BLASTP from collected genomes with AtMS1, AtMMD1, and AT1G33420 as query sequences. A total of 116 sequences were aligned using MUSCLE v3.8 (Edgar 2004). The resultant alignments (Supplementary Data Set 8) were used to construct a maximum likelihood phylogenetic tree using RAxMLv8 (Stamatakis 2014) with the PROTGAMMAAUTO model to calculate the support value with 1,000 bootstrap replicates. The phylogenetic tree was visualized with FigTree V1.4.4 (http://tree.bio.ed.ac.uk/software/figtree/).

### Statistical analysis

Significant differences were assessed by unpaired 2-tailed Student's *t* test using GraphPad Prism Version 8. See Supplementary Data Set 9 for the summary of statistical tests.

### Accession numbers

Sequence data from this article can be found at the MarpolBase (https://marchantia.info/) or TAIR (https://www.arabidopsis.org/) by following accession numbers Mp*GLID* (Mp3g17000), Mp*BNB* (Mp3g23300), and *AtMS1* (AT5G22260). *Marchantia polymorpha* genes referred in RNA-seq analysis are listed in Supplementary Data Sets 2, 3, and 5.

## Supplementary Material

koae206_Supplementary_Data

## Data Availability

Raw data for transcriptome analysis generated in this study have been deposited at China National GeneBank DataBase (CNGBdb) under project CNP0005192.

## Supplementary data

The following materials are available in the online version of this article.

**Supplementary Figure S1.** Abundance of the Mp*GLID* transcripts in various tissues of *M. polymorpha*.

**Supplementary Figure S2.** Schematic illustration of the plasmids used to generate transgenic *M. polymorpha*.

**Supplementary Figure S3.** No discernable differences observed at the macroscopic level between the WT and Mp*glid^ge^* mutants.

**Supplementary Figure S4.** Antheridiophores from the WT and Mp*glid^ge^* mutants.

**Supplementary Figure S5.** Number of antheridia in antheridiophores from the WT and Mp*glid*^ge^ mutants.

**Supplementary Figure S6.** Generation of transgenic *M. polymorpha* plants overexpressing Mp*GLID*.

**Supplementary Figure S7.** Observation of female thalli overexpressing Mp*GLID*.

**Supplementary Figure S8.** Genes predominantly expressed in developing antheridia were induced by overexpression of Mp*GLID* in thalli.

**Supplementary Figure S9.** Induction of antheridiophores by overexpression of Mp*BNB*.

**Supplementary Figure S10.** Identification of *pro*Mp*EF1:*Mp*BNB*/Mp*glid^ge^* plants.

**Supplementary Figure S11.** Controls used in the yeast 1-hybrid assay.

**Supplementary Figure S12.** No sporophore formed in the Mp*glid^ge^* mutant.

**Supplementary Figure S13.** Evolutionary analysis of the MpGLID homologs.

**Supplementary Figure S14.** Multiple sequence alignment for the PHD finger domains of group B members in land plants.

**Supplementary Figure S15.** Domain-swapping analysis between MpGLID and AtMS1.

**Supplementary Figure S16.** Functional analysis of Mp3g06700 in *M. polymorpha* male gametogenesis.

**Supplementary Figure S17.** Chromatic expression of AT1G33420 in the *A. thaliana* eFP browser.

**Supplementary Figure S18.** Detection of the Mp*GLID* promoter activities during sporophyte development.

**Supplementary Data Set 1.** Quality of RNA-seq data used in this study.

**Supplementary Data Set 2.** List of genes specifically expressed in antheridia at early stage.

**Supplementary Data Set 3.** Genes with higher expression levels in antheridia at early stage compared with middle or late stages.

**Supplementary Data Set 4.** Five TFs are specifically expressed at high level in antheridia at early stage.

**Supplementary Data Set 5.** RNA-seq data for transgenic *M. polymorpha* constitutively overexpressing Mp*GLID*.

**Supplementary Data Set 6.** Seventy-two genes with specific high expression in antheridia at early stage are significantly upregulated by the overexpression of Mp*GLID* in *M. polymorpha* thalli.

**Supplementary Data Set 7.** List of primers used in this study.

**Supplementary Data Set 8.** Alignments used for phylogenetic construction.

**Supplementary Data Set 9.** Summary of statistical tests in this study.
