# Supplementary material for: The BNB–GLID module regulates germline fate determination in Marchantia polymorpha
Source: Plant Cell. 2024 Jul 23;36(9):3824–37. doi: 10.1093/plcell/koae206 (PMC11371191; doi:10.1093/plcell/koae206)
Supplement: koae206_Supplementary_Data [file koae206_supplementary_data.zip › TPC2024RA00075R2_Supplementary_Figures.pdf]

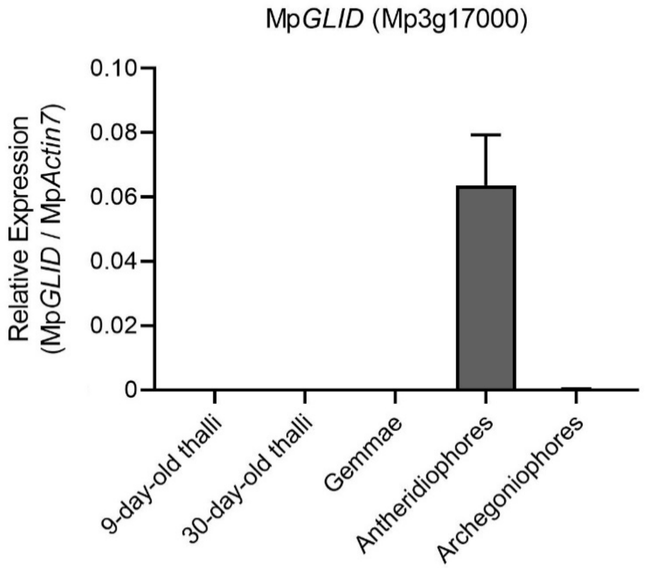

Supplementary Figure S1. Abundance of Mp*GLID* transcripts in various tissues of *M. polymorpha*. Quantitative RT-PCR analysis shows predominant expression of Mp*GLID* in antheridiophores. Antheridiophores and archegoniophores 28 days after reproductive induction were collected. Mp*Actin7* was used as an internal control. Bars represents mean measurements ± SD (n=3).

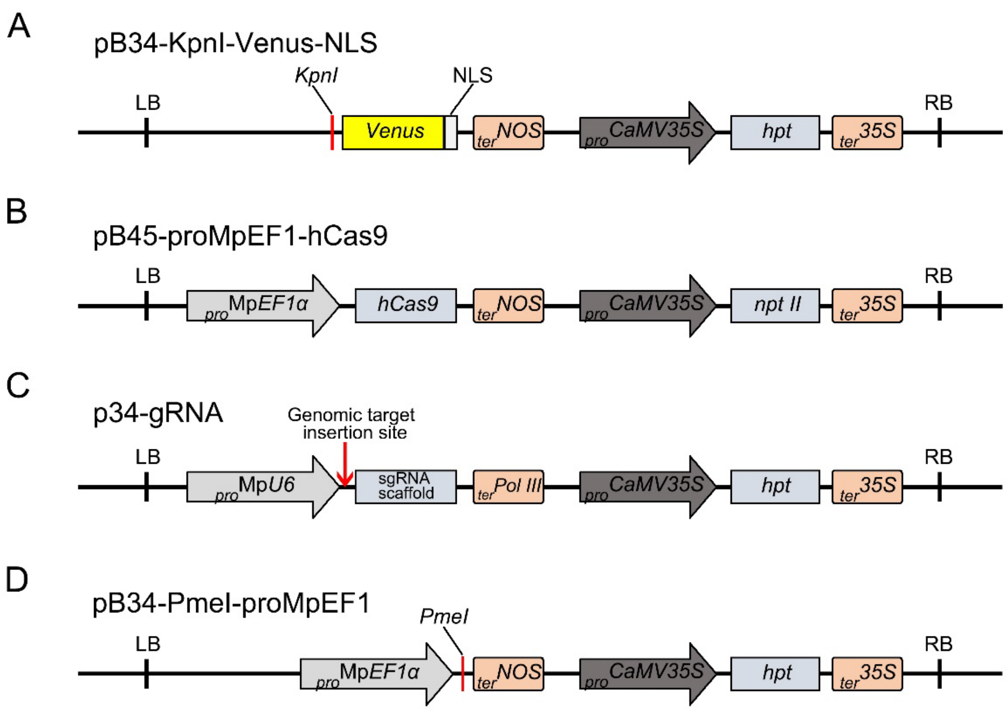

Supplementary Figure S2. Schematic illustration of plasmids used to generate transgenic *M. polymorpha*. A) Plasmid pB43-KpnI-Venus-NLS for expression pattern analysis, harboring a hygromycin-resistance cassette driven by CaMV35S promoter for transgenic plant selection. The restriction enzyme *Kpn*I is used to linearize the plasmid. **B**) Plasmid pB45-proMpEF1-hCas9 for overexpression of hCas9 protein, harboring a kanamycin-resistance cassette driven by CaMV35S promoter for transgenic plant selection. **C**) Plasmid p34-gRNA for overexpression of gRNA, harboring the hygromycin-resistance cassette driven by CaMV35S promoter for transgenic plant selection. Red arrow indicates the gRNA sequence insertion site. **D**) Plasmid pB34-PmeI-ProMpEF1 for constitutive overexpression analysis, harboring a hygromycin-resistance cassette driven by CaMV35S promoter for transgenic plant selection. The restriction enzyme *Pme*I is used to linearize the plasmid.

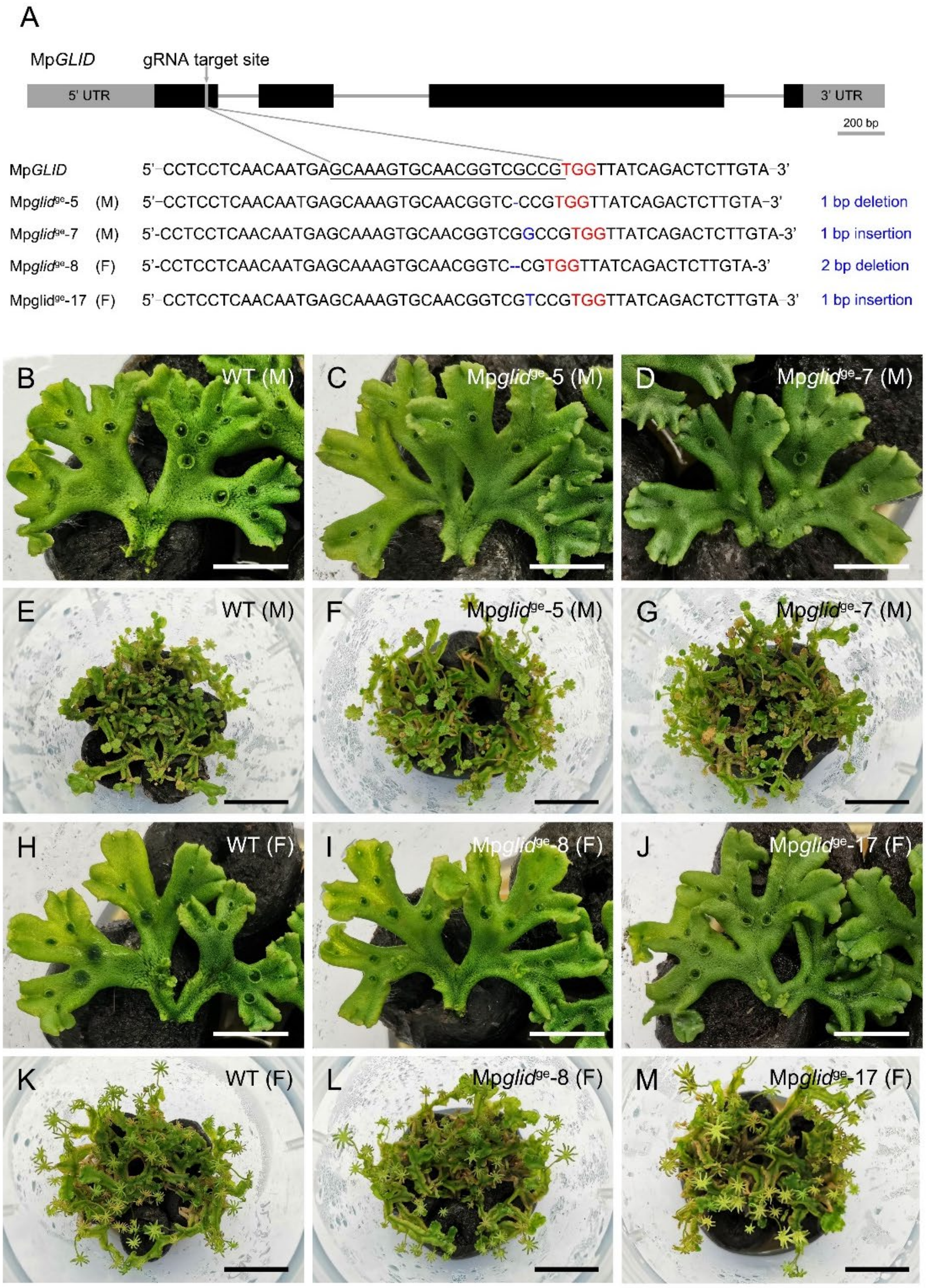

Supplementary Figure S3. No discernable differences observed at macroscopic level between wild type and Mp*glid*^{ge} mutants. A) Schematic illustration of Mp*GLID* gene structure and mutations identified in the Mp*GLID* locus. Gray and black boxes indicate untranslated regions (UTR) and exons, respectively. Lines in gray between exons represent introns. The gRNA targeting site is indicated by a gray arrow. The DNA sequence of protospacer adjacent motif (PAM) is indicated in red. M, male; F, female. **B-M**) Thalli of wild type (**B**, **H**) and Mp*glid*^{ge} mutants (**C**, **D**, **I-J**) 21 days after transplantation. Reproductive growth of wild type (**E**, **K**) and Mp*glid*^{ge} mutants (**F**, **G**, **L-M**) 40 days after induction. No differences were observed in vegetative (**B-D**, **H-J**) or reproductive growth (**E-G**, **K-M**). M, male; F, female. Scale bars, 2 cm (**B-D**, **H-J**); 4 cm (**E-G**, **K-M**).

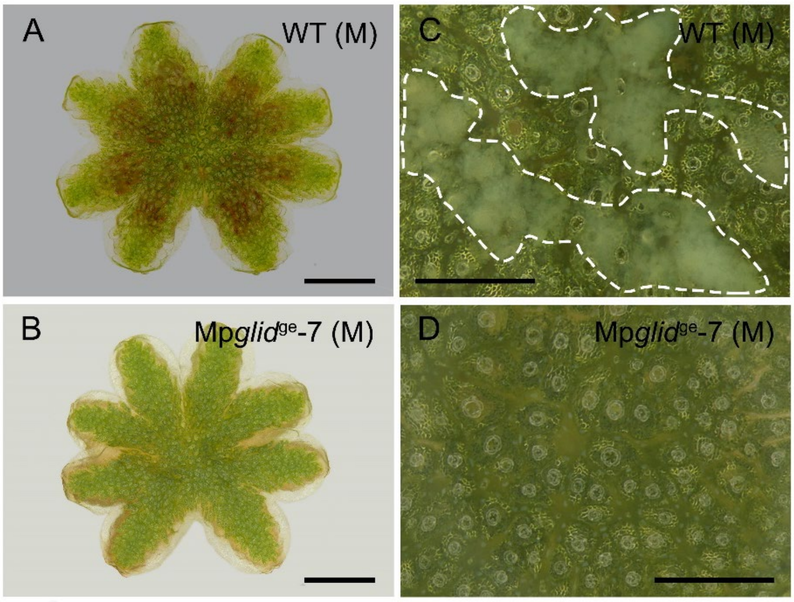

Supplementary Figure S4. Antheridiophores from wild type and Mp*glid*^{ge} mutant. A, **B**) Antheridiophores from wild type (**A**) and Mp*glid*^{ge} mutant (**B**) 40 days after reproductive induction. Scale bars, 4 mm. **C**, **D**) Sperm discharged from mature antheridia in wild type plants (**C**) and Mp*glid*^{ge}-7 mutant (**D**) when water was applied to the top of antheridiophores. Dashed lines in white indicate sperm clouds released from antheridia of a wild type plant (**C**), but no sperm clouds were observed for the Mp*glid*^{ge}-7 mutant (**D**). M, male. Scale bars, 1mm.

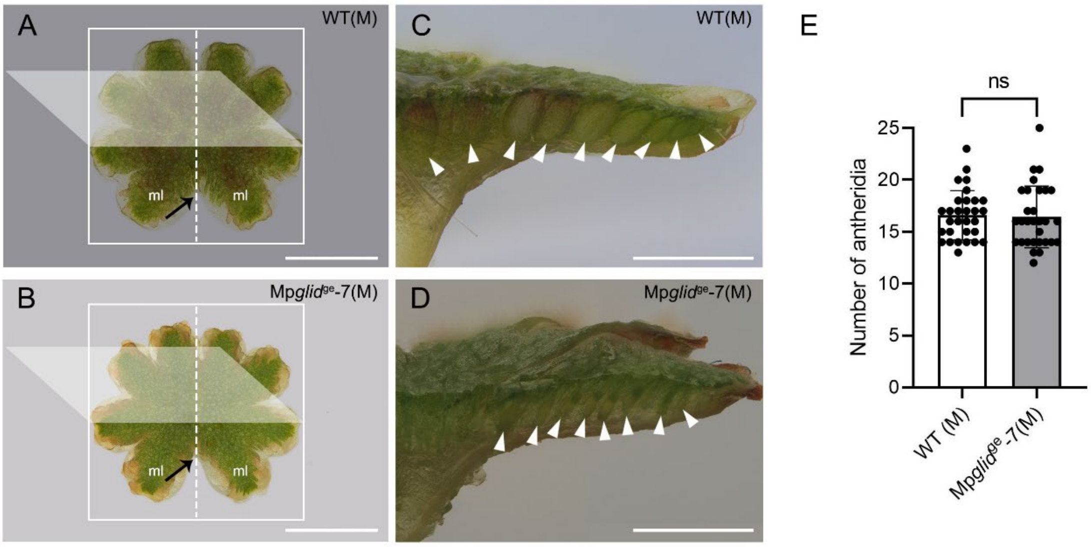

Supplementary Figure S5. Number of antheridia in antheridiophores from wild type and Mp*glid*^{ge} mutant. A, **B)** Illustration of transverse section of antheridiophores from wild type (**A**) and Mp*glid*^{ge}-7 mutant (**B**) 28 days after reproductive induction. The disk-like receptacles usually comprise 8 lobes, with an opening (black arrows) formed by two margin lobes (ml). Dashed lines indicate the longitudinal axis of the receptacle, and transverse sections were made perpendicularly to the longitudinal axis. Scale bars, 6 mm. **C**, **D**) Transverse sections of receptacles from wild type (**C**) and Mp*glid*^{ge}-7 mutant (**D**). White arrowheads indicate the antheridia. Scale bars, 2 mm. **E**, Bar chart showing no difference in the number of antheridia between wild type and Mp*glid*^{ge}-7 mutant. ns, not significant (n=30, Student's *t*-test); M, male.

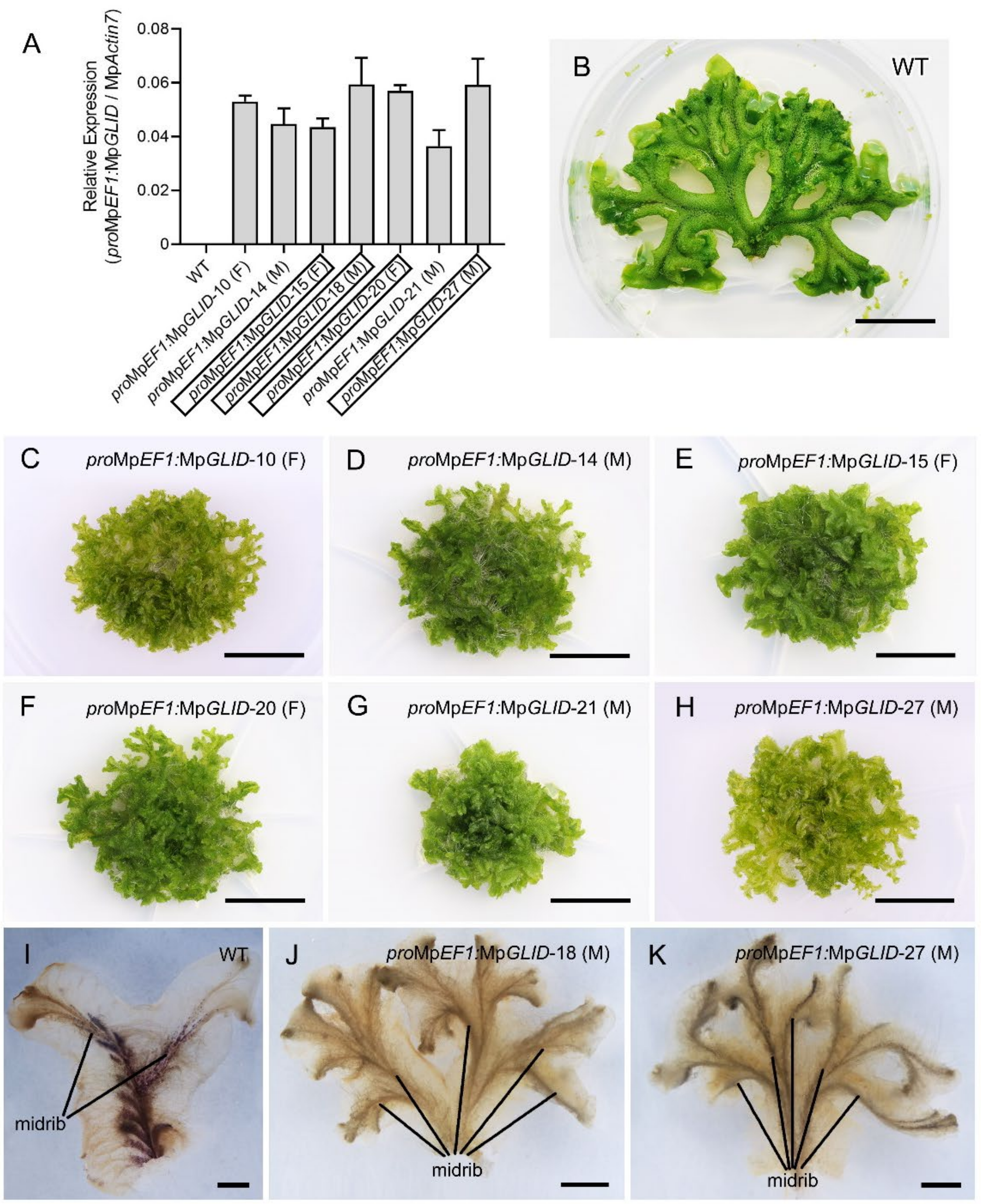

Supplementary Figure S6. Generation of transgenic *M. polymorpha* plants overexpressing Mp*GLID*. A) Relative abundance of Mp*GLID* transcripts in thalli of wild type and *pro*Mp*EF1a:*Mp*GLID* plants 28 days after transplantation. Mp*Actin7* was selected as internal control. Bars represents mean measurements ± SD (n=3). Four independent transgenic lines including two male (M) and two female (F) indicated by black boxes were selected for further analysis in this study. **B-H**) Top view of thalli from wild type (**B**) and *pro*Mp*EF1a:*Mp*GLID* plants (**C-H**) 28 days after transplantation. Scale bars, 2 cm. **I-K**) Ventral view of thalli from wild type (**I**) and *pro*Mp*EF1a:*Mp*GLID* plants (**J**, **K**). Chlorophyll was removed with a 70% ethanol solution (w/v). Scale bars, 4mm.

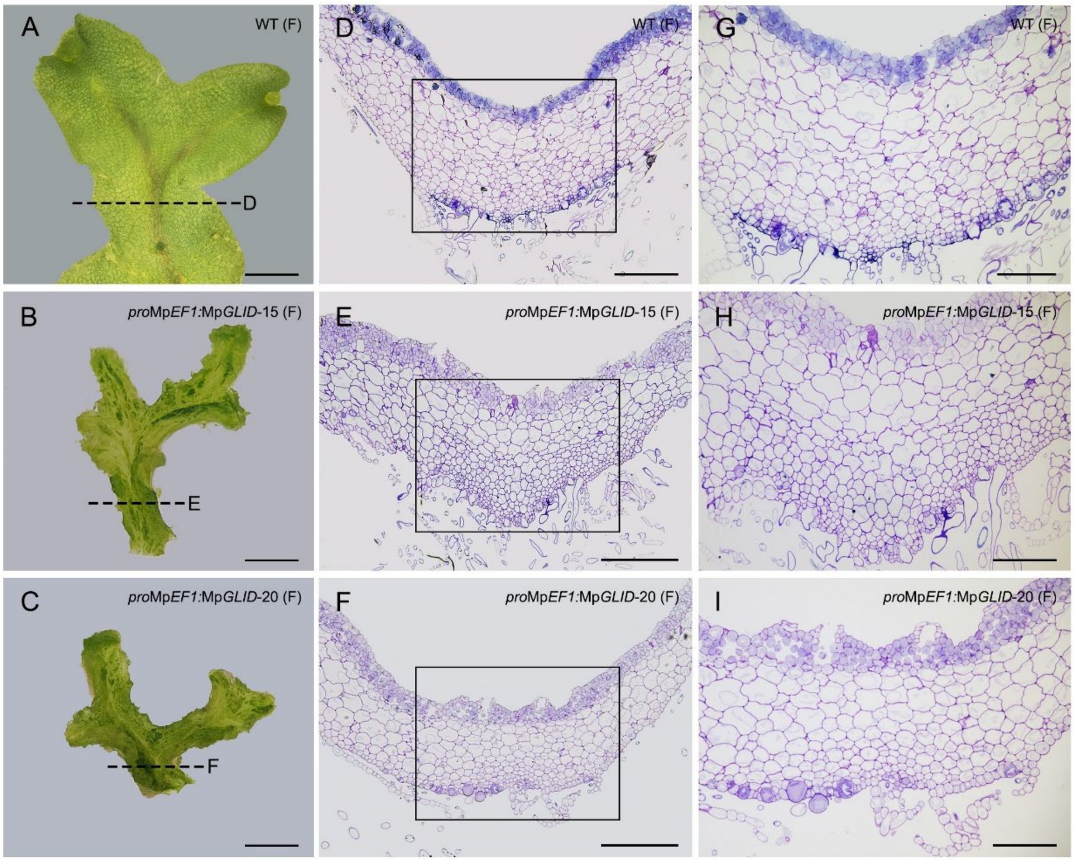

Supplementary Figure S7. Observation of female thalli overexpressing Mp*GLID*. A-**C**) Dorsal views of wild type (**A**), *pro*Mp*EF1a*:Mp*GLID*-15 (**B**), and *pro*Mp*EF1a*:Mp*GLID*-20 (**C**) 21 days after transplantation. The positions of transverse sections of thalli are indicated by black dotted lines. F, Female; Scale bars, 0.5 cm. **D-F**) Transverse sections of thalli from wild type (**D**) and female plants overexpressing Mp*GLID* gene (**E**, **F**). Transverse sections from the positions indicated in (**A-C**) respectively. Scale bars, 400 μm. **G-I**) Close-up views highlighted by black boxes in (**D-F**) respectively. Scale bars, 200 μm.

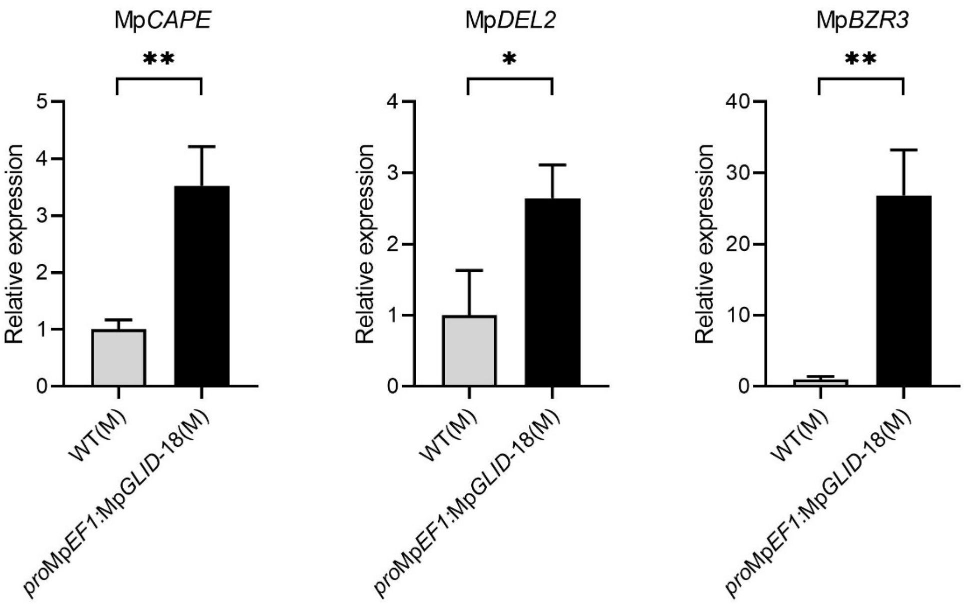

Supplementary Figure S8. Genes predominantly expressed in developing antheridia were induced by overexpression of Mp*GLID* in thalli. Gray and black bars indicate wild-type (WT) and *pro*Mp*EF1*:Mp*GLID*, respectively. M, male. Bars represents mean measurements ± SD. * $P < 0.05$ and ** $P < 0.01$ (n=3, Student's *t*-test).

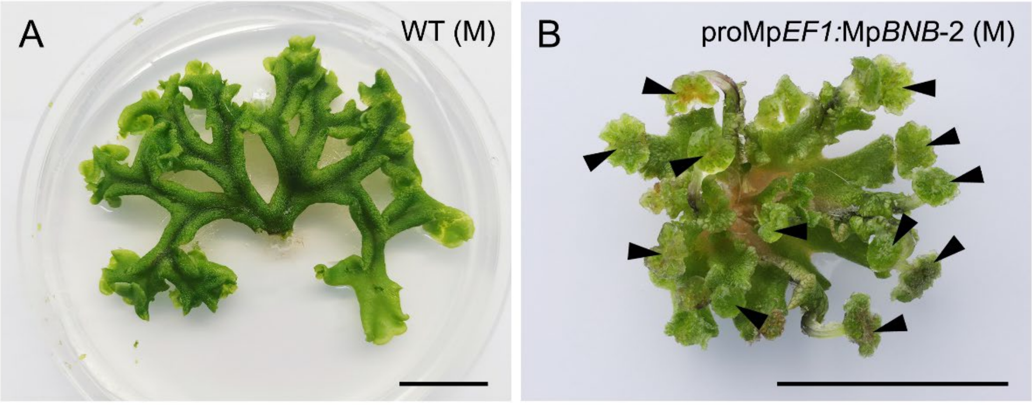

Supplementary Figure S9. Induction of antheridiophores by overexpression of Mp*BNB*. A) A representative wild type plant. **B**) A representative transgenic plant overexpressing Mp*BNB*. Plants were cultured on 1/2 GB media for 28 days after transplantation under normal vegetive growth conditions. Arrowheads in black indicate antheridiophores. Scale bars, 4 cm (A); 2 cm (B).

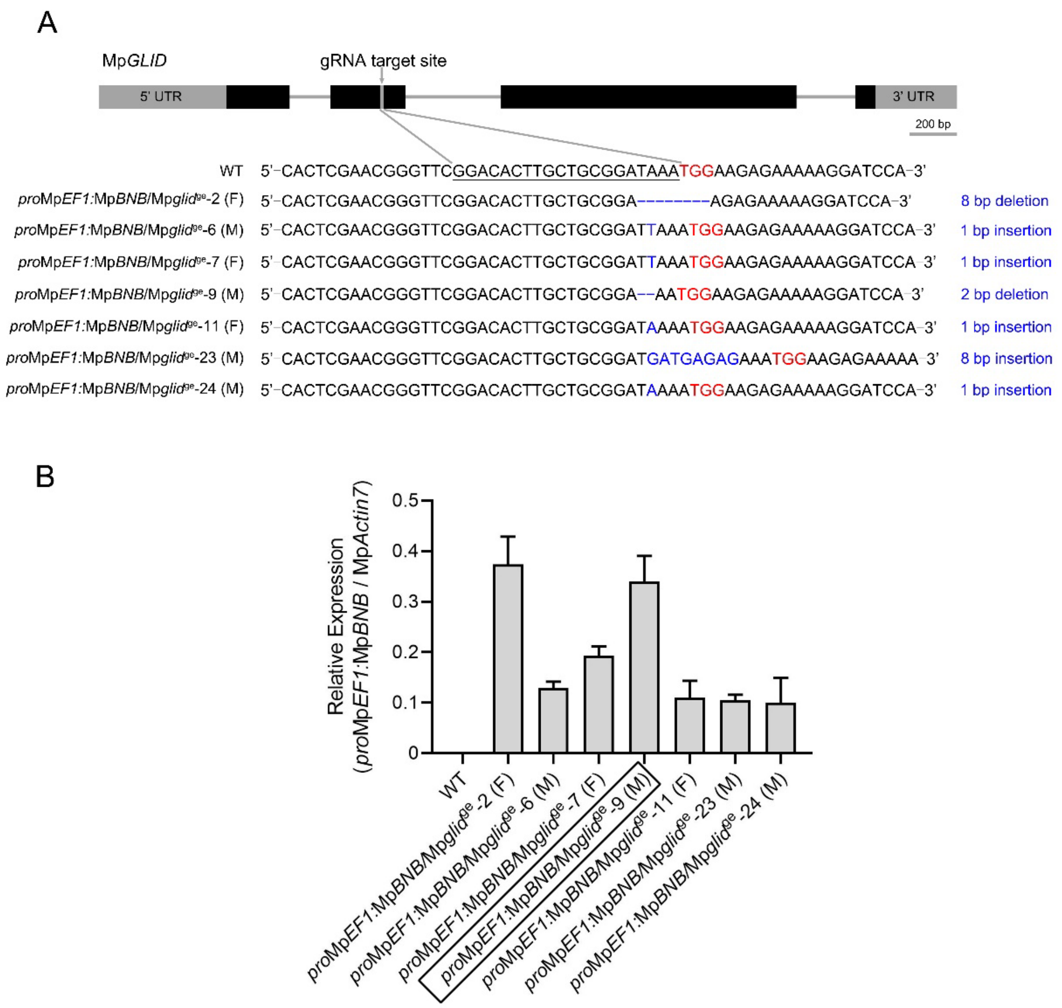

Supplementary Figure S10. Identification of *pro*Mp*EF1:*Mp*BNB*/Mp*glid^{ge}* plants. A) Schematic illustration of Mp*GLID* gene structure and mutations identified in the Mp*GLID* locus. Gray and black boxes indicate the untranslated regions (UTR) and exons, respectively. Lines in gray between exons represent introns. The gRNA targeting site is indicated by a gray arrow. The DNA sequence of protospacer adjacent motif (PAM) is indicated in red. Inserted DNA sequence is indicated in blue. M, male; F, female. **B**) Relative abundance of overexpressed Mp*BNB* transcripts in 28-day-old thalli of wild-type (WT) and transgenic *pro*Mp*EF1:*Mp*BNB*/Mp*glid^{ge}* plants. Mp*Actin7* was selected as the internal control. *pro*Mp*EF1:*Mp*BNB*/Mp*glid^{ge}*-9 (highlighted by black box) was selected for further analysis in this study. M, male; F, female. Bar measurements represent mean measurements ± SD (n=3).

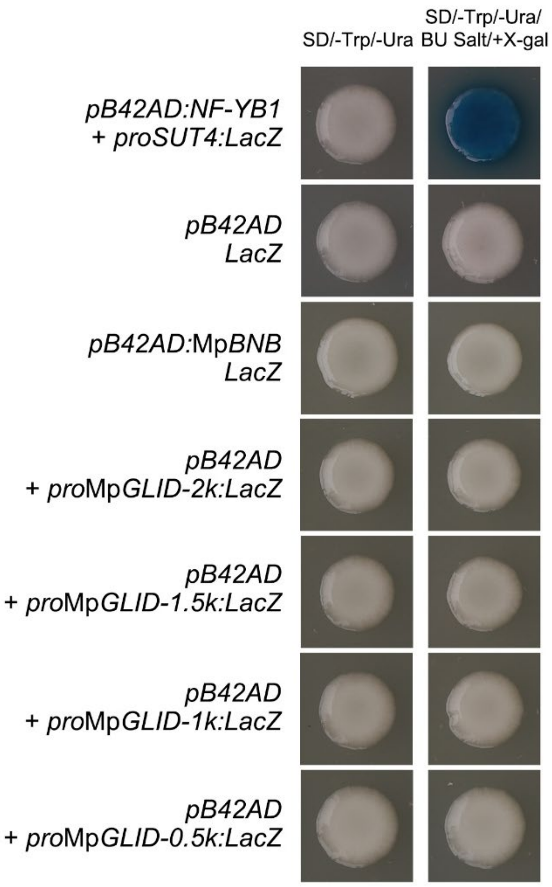

Supplementary Figure S11. Controls used in yeast one-hybrid assay. Yeast cells were co-transformed with indicated plasmids and cultured on SD/-Trp/-Ura medium supplemented with X-gal. NF-YB1 and *OsSUT4* promoter sequence were used as positive control.

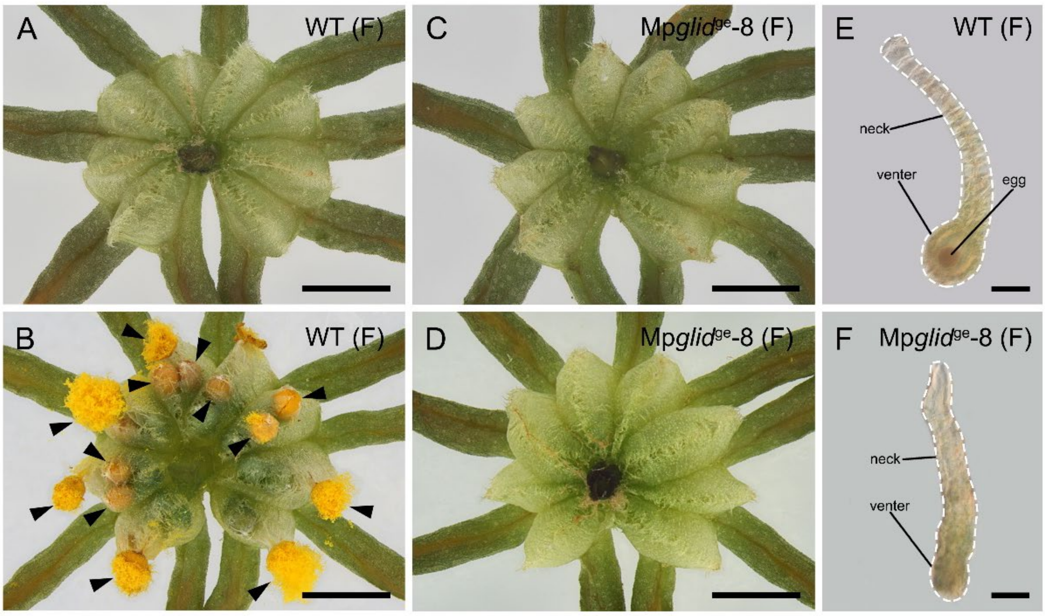

Supplementary Figure S12. No sporophore formed in Mp*glid*^{ge} mutant. A-D) Ventral view of archegoniophores from the wild type (**A**, **B**) and Mp*glid*^{ge}-8 mutant (**C**, **D**) before (**A**, **C**) and after (**B**, **D**) crossing with wild type male plants. Black arrows indicate mature sporophores of a wild type plant, but no sporophores were formed in the Mp*glid*^{ge}-8 mutant 28 days after crossing. Scale bars, 4mm. **E**, **F**) Archegonia from wild type (**E**) and Mp*glid*^{ge}-8 mutant (**F**). No egg cell was defined and the venter failed to swell. Scale bars, 50 µm.

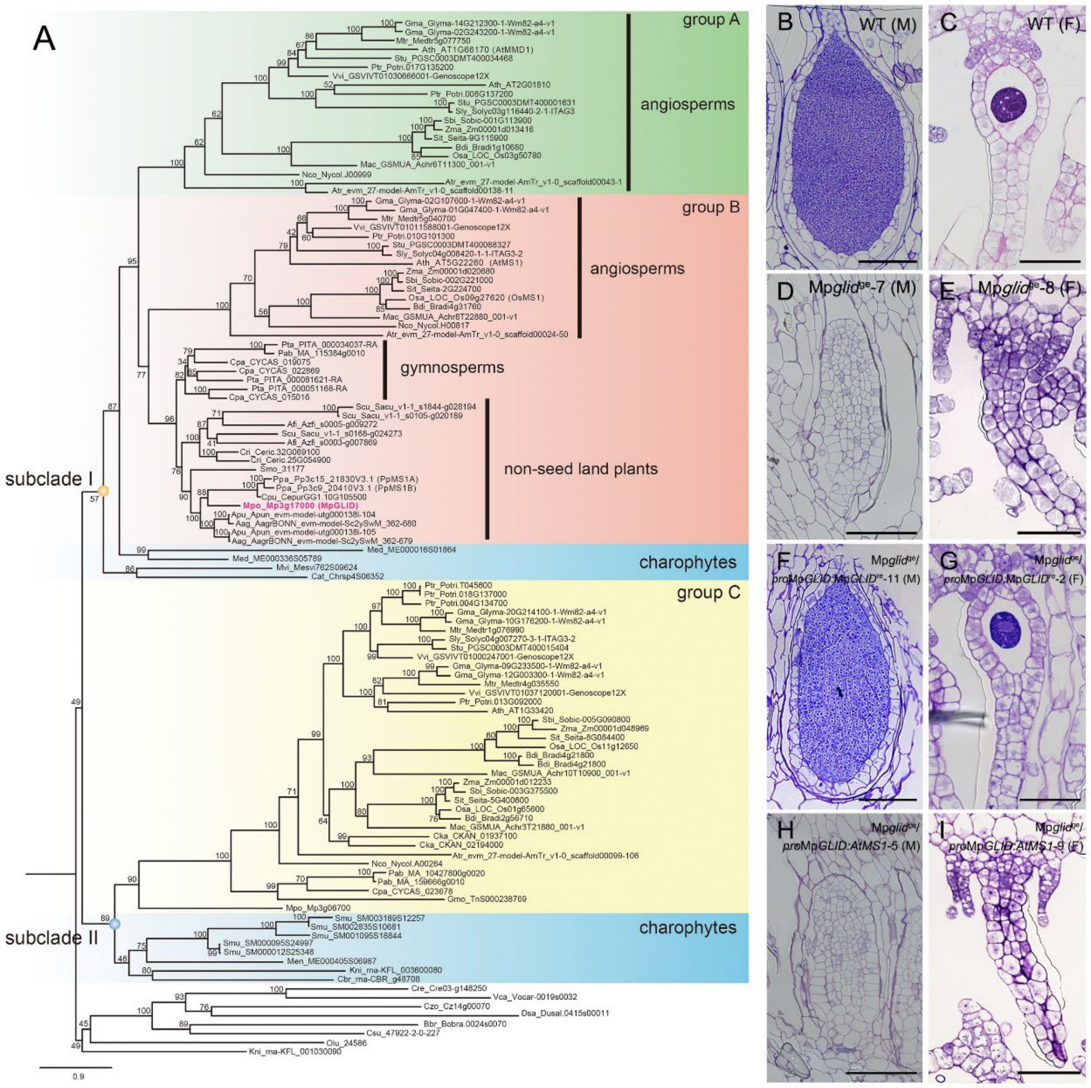

Supplementary Figure S13. Evolutionary analysis of MpGLID homologues. **A**) Phylogenetic tree of MpGLID homologues across the green plant kingdom. A total of 116 amino acid sequences of putative proteins homologous to MpGLID were identified from 36 representative plant species from green algae to angiosperms. Values at each branches indicate bootstrap values calculated with 1,000 replicates. The scale bar represents the amino acid divergence per site. Olu, *Ostreococcus lucimarinus*; Csu, *Coccomyxa subellipsoidea*; Bra, *Botryococcus braunii*; Dua, *Dunaliella salina*; Cre, Chl*amydomonas reinhardtii*; Vca, *Volvox carteri*; Czo, *Chromochloris zofingiensis*; Cat, *Chlorokybus atmophyticus*; Atr, *Amborella trichopoda*; *Pta, Pinus taeda*; Cpa, *Cycas panzhihuaensis*; Mvi, *Mesostigma viride*; Kni, *Klebsormidium nitens*; Cbr, *Chara braunii*; Smu, *Spirogloea muscicola*; Men, *Mesotaenium endlicherianum*; Aag, *Anthoceros agrestis*; Apu, *Anthoceros punctatus*; Mpo, *Marchantia polymorpha*; Cpu, *Ceratodon purpureus*; Ppa, *Physcomitrium patens*; Smo, *Selaginella moellendorffii*; Cri, *Ceratopteris richardii*; Afi, *Azolla filiculoides*; Scu, *Salvinia trichopoda*; Nco, *Nymphaea colorata*; Mac, *Musa acuminata*; Bdi, *Brachypodium distachyon*; Ost, *Oryza sativa*; Sit, *Setaria italica*; Sbi, *Sorghum bicolor*; Zma, *Zea mays*; Ath, *Arabidopsis thaliana*; Mtr, *Medicago truncatula*;

Gma, *Glycine max*; Vvi, *Vitis vinifera*; Ptr, *Populus trichocarpa*; Sly, *Solanum lycopersicum*; Stu, *Solanum tuberosum*. **B-I**) Cross sections of antheridia (**B**, **D**, **F, H**) and archegonia (**C**, **E**, **G, I**) from wild type (**B**, **C**), Mp*glid*^{ge} (**D**, **E**), *pro*Mp*GLID*: Mp*GLID*^{re}/Mp*glid*^{ge} (**F**, **G**), and *pro*Mp*GLID*:*AtMS1* Mp*glid*^{ge} (**H**, **I**). Scale bars, 100 µm (**B, F**); 50 µm (**C-E, G-I**).

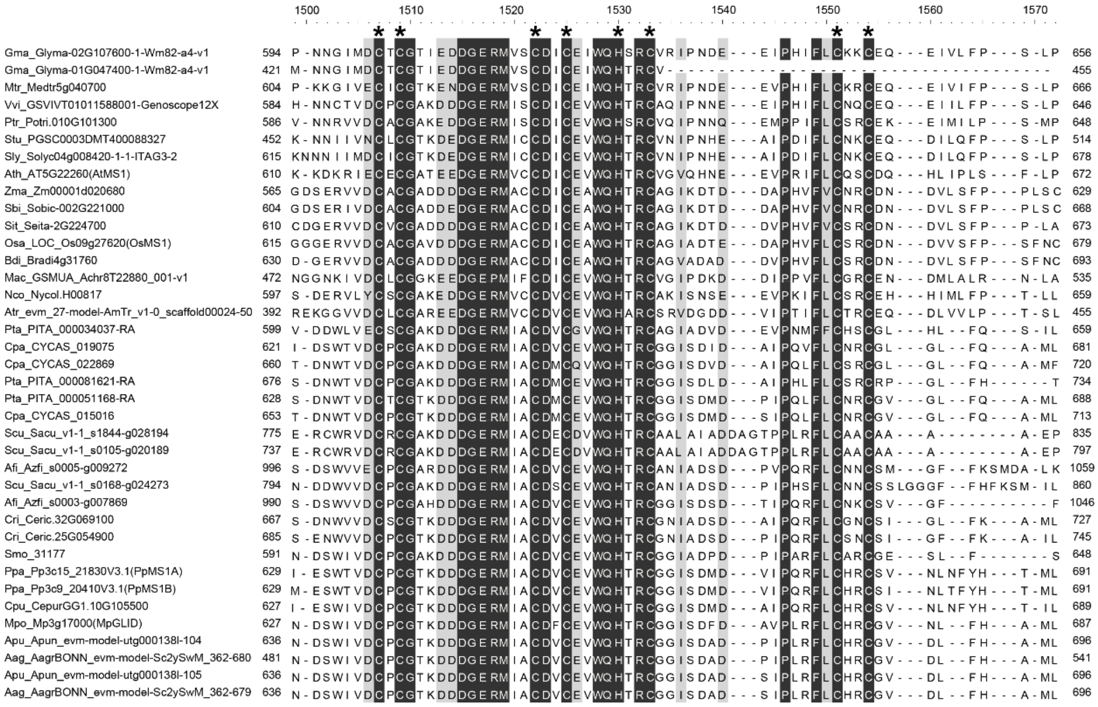

Supplementary Figure S14. Multiple sequence alignment for PHD-finger domains of group B members in land plants. The group B members possess a typical PHD-finger domain with a characteristic core Cys4-His-Cys3 amino acid organization (indicated by asterisks) structural arrangement. Gma, *Glycine max*; Mtr, *Medicago truncatula*; Vvi, *Vitis vinifera*; Ptr, *Populus trichocarpa*; Stu, *Solanum tuberosum*; Sly, *Solanum lycopersicum*; Ath, *Arabidopsis thaliana*; Zma, *Zea mays*; Sbi, *Sorghum bicolor*; Sit, *Setaria italica*; Osa, *Oryza sativa*; Bdi, *Brachypodium distachyon*; Mac, *Musa acuminata*; Nco, *Nymphaea colorata*; Atr, *Amborella trichopoda*; *Pta, Pinus taeda*; Cpa, *Cycas panzhihuaensis*; Scu, *Salvinia trichopoda*; Afi, *Azolla filiculoides*; Cri, *Ceratopteris richardii*; Smo, *Selaginella moellendorffii*; Ppa, *Physcomitrium patens*; Cpu, *Ceratodon purpureus*; Mpo, *Marchantia polymorpha*; Apu, *Anthoceros punctatus*; Aag, *Anthoceros agrestis*.

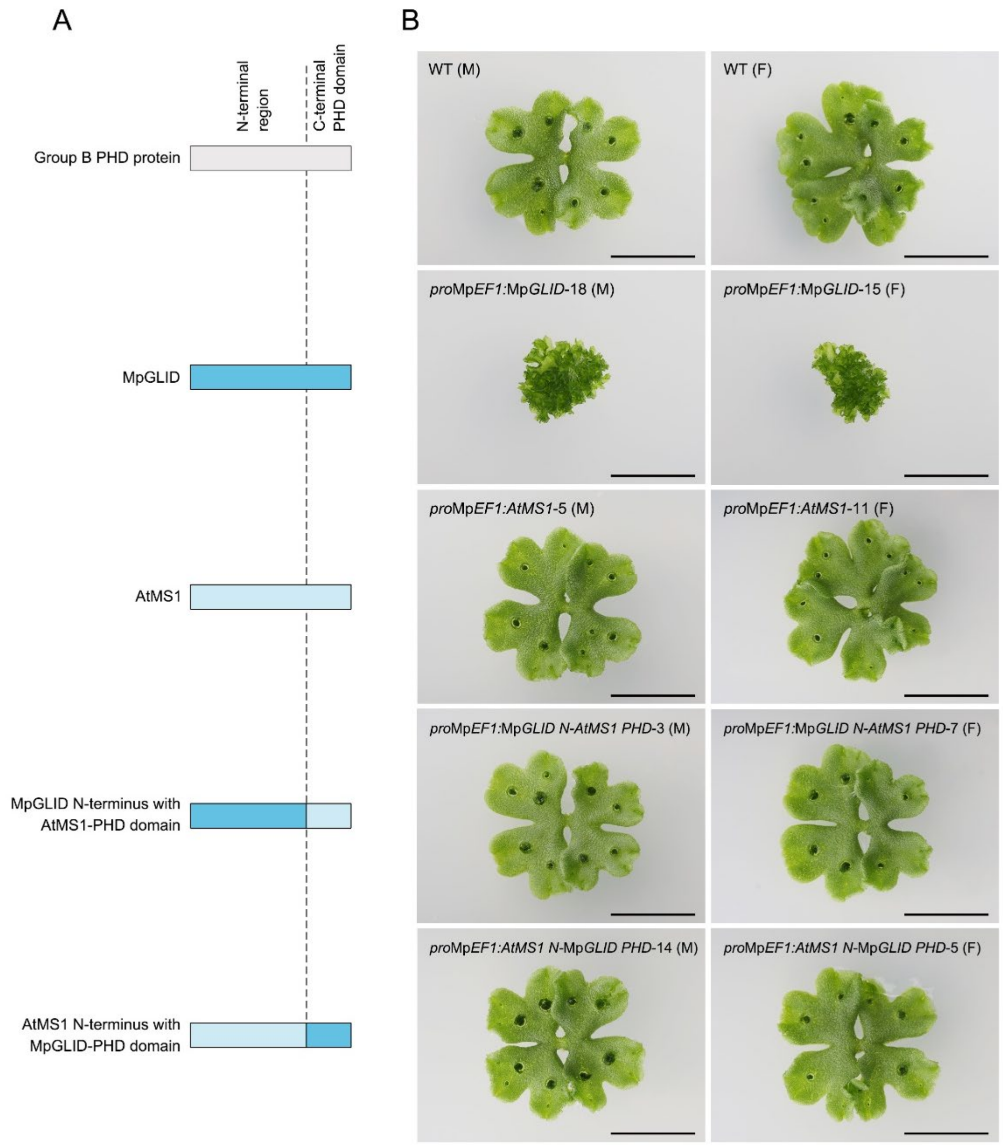

Supplementary Figure S15. Domain-swapping analysis between MpGLID and AtMS1. A) Schematic illustration of constructing chimeric constructs. **B**) Phenotypes observations of 14-day-old wild-type (WT) and transgenic plants ectopically overexpressing chimeric proteins. M, male; F, female. Scale bars, 1 cm.

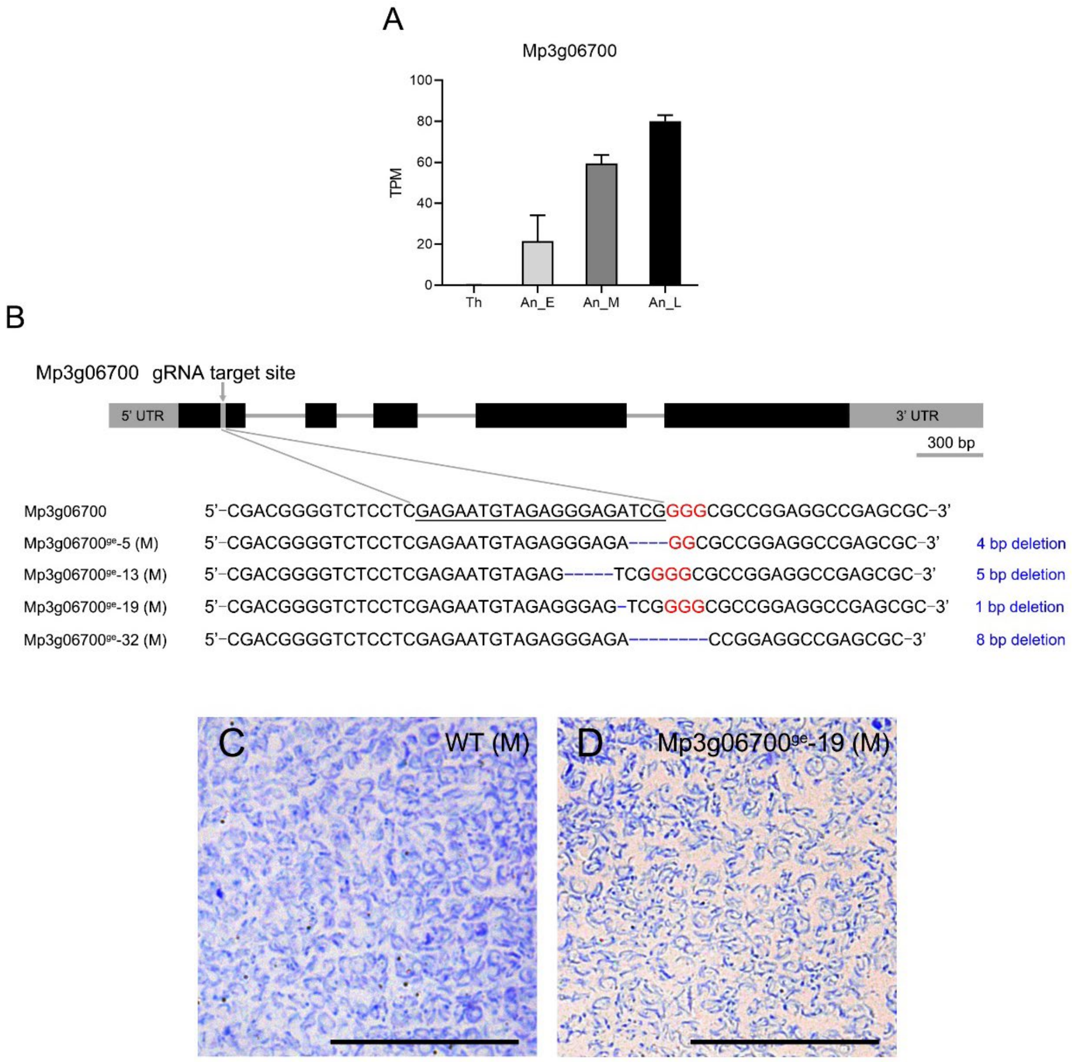

Supplementary Figure S16. Functional analysis of Mp3g06700 in *M. polymorpha* male gametogenesis. A) Expression of Mp3g06700 during antheridium development based on RNA-seq analysis. Th, thallus; An_E, antheridia at early stage; An_M, antheridia at middle stage; An_L, antheridia at late stage. Bars represents mean expression ± SD (n=4). **B**) Schematic illustration of Mp*GLID* gene structure and mutations identified in the Mp3g06700 locus. Gray and black boxes indicate the untranslated regions (UTR) and exons, respectively. Lines in gray between exons represent introns. The gRNA targeting site is indicated by a gray arrow. The DNA sequence of protospacer adjacent motif (PAM) is indicated in red. M, male. The line in bold was selected for further analysis. **C, D**) Differentiated sperm cells in wild-type (WT) plants and Mp3g06700 mutants. No differences were observed. Scale bars, 50 µm.

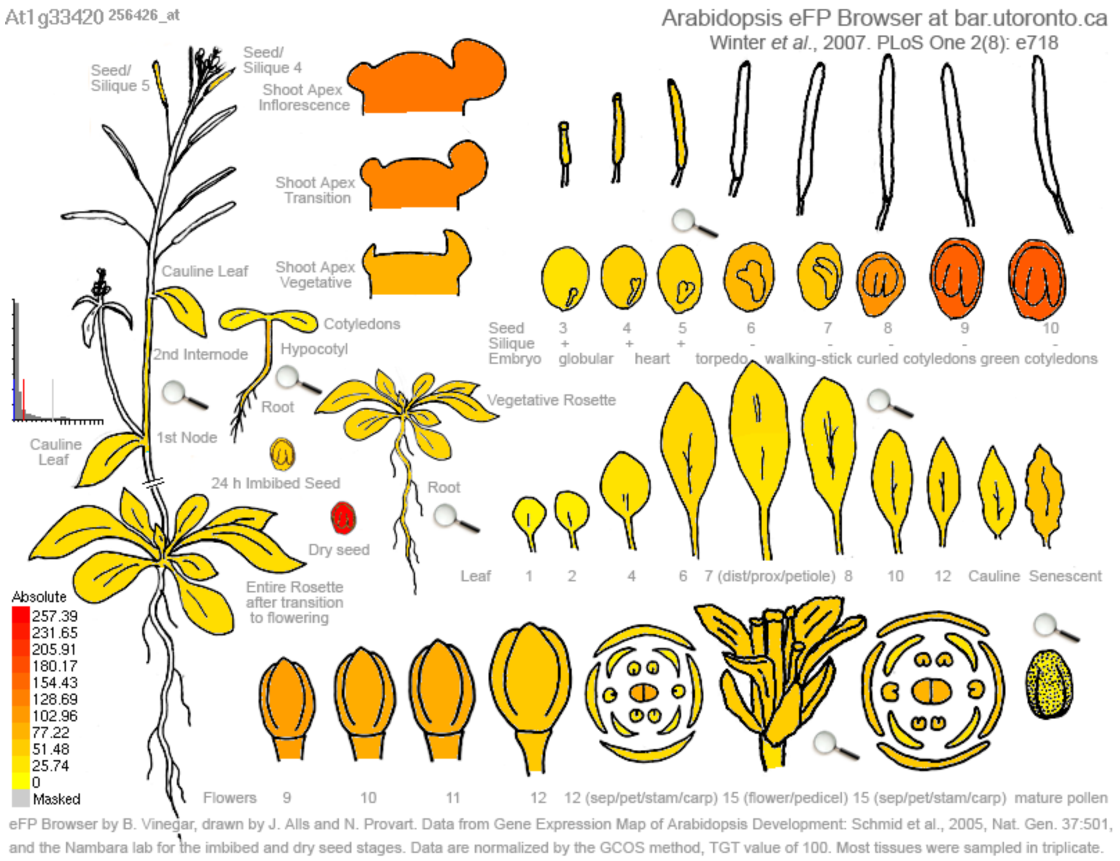

Supplementary Figure S17. Chromatic expression of AT1G33420 in the *Arabidopsis thaliana* eFP browser.

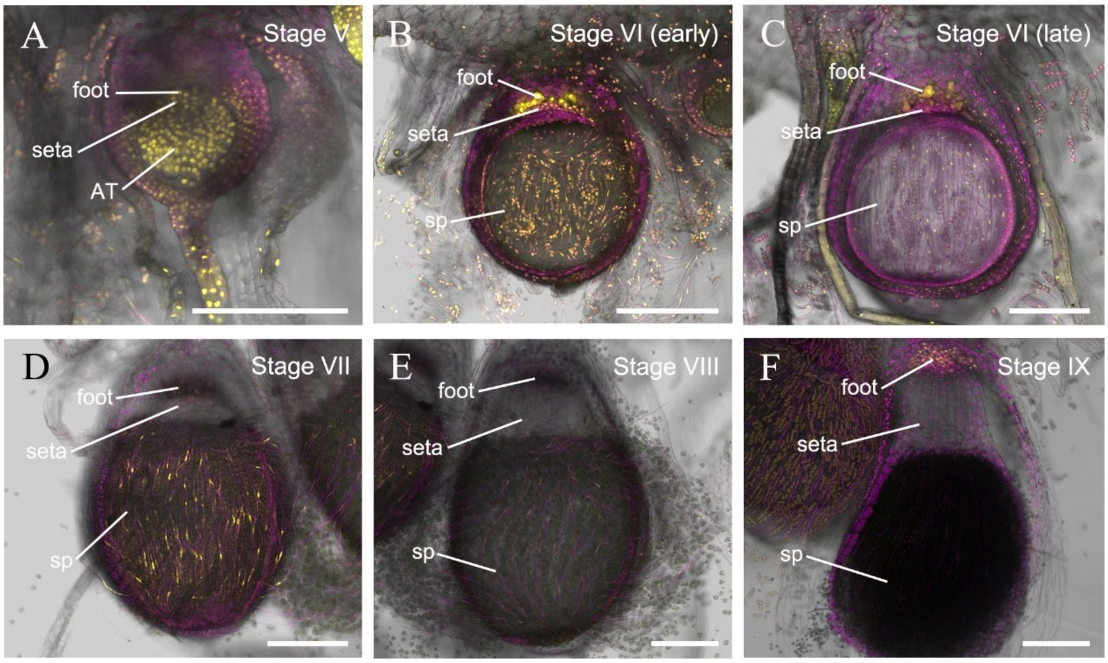

Supplementary Figure S18. Detection of Mp*GLID* promoter activities during sporophyte development. Accumulation of Venus-NLS proteins in various developmental stages of sporophytes. Sporophytes were induced by crossing male and female transgenic *pro*Mp*GLID:Venus-NLS* plants, in which the expression of Venus-NLS proteins was under control of the Mp*MAGS* promoter. The stages of sporophyte development were defined by following Moriya et al. (2023). Yellow and magenta fluorescence indicate Venus-NLS and autofluorescence respectively. AT, archesporial tissue; sp, sporangium. Scale bars, 400 µm (**A**); 50 µm (**B-F**).

Moriya KC, Shirakawa M, Loue-Manifel J, Matsuda Y, Lu YT, Tamura K, Oka Y, Matsushita T, Hara-Nishimura I, Ingram G, et al. (2023) Stomatal regulators are co-opted for seta development in the astomatous liverwort *Marchantia polymorpha*. Nat Plants **9**(2): 302-314.
