# Supplementary material for: The BNB–GLID module regulates germline fate determination in Marchantia polymorpha
Source: Plant Cell. 2024 Jul 23;36(9):3824–37. doi: 10.1093/plcell/koae206 (PMC11371191; doi:10.1093/plcell/koae206)
Supplement: koae206_Supplementary_Data [file tpc.00075.2024-s07.pdf › tpc.00075.2024-s07.pdf]

THE PLANT CELL

The genetic module BNB-GLID regulates germline fate determination in the liverwort Marchantia polymorpha

Xiaolong Ren, Xiaoxia Zhang, Xiaotong Qi, Tian Zhang, Huijie Wang, David Twell, Yu Gong, Yuan Fu, Bai-Chen Wang, Hongzhi Kong, and Bo Xu

Review Timeline:

Submission Date:	02-Feb-2024
Editorial Decision:	24-Feb-2024
Revision Received:	29-Apr-2024
Editorial Decision:	24-May-2024
Revision Received:	05-Jun-2024
Accepted:	06-Jun-2024

Dr. Bo Xu
Institute of Botany Chinese Academy of Sciences
Beijing
China

Dear Dr. Xu:

We have received reviews of your manuscript entitled "Rewiring of a genetic module regulating germline fate determination during land plant evolution." Thank you for submitting your best work to The Plant Cell. The editorial board agrees that the work you describe is substantive, falls within the scope of the journal, and may become acceptable for publication, pending revision and re-review.

The reviewers and editorial board are excited about the work deciphering a part of the germline specification GRN in Marchantia and its evolutionary perspective. The molecular and functional links between MpBNB and MpGLID are clear; however, the evolution of the BNB-GLID GRN in land plants lacks significant components to support the authors' perspective. GLID and BNB genes, or related genes, have already been studied and reported elsewhere. Therefore, the evolutionary perspective of the GLID-BNB GRN becomes essential.

We ask you to pay attention to the following points in preparing your revision:

1. Paralogs in both Marchantia (MpGLID and Mp3g06700) and Arabidopsis (MS1 and At1g33420) are present. Further characterization of these paralogous genes are critical to explore the evolutionary conservation and shift.

2. AtMS1 does not complement the Mpglid mutant phenotype, and it is not clear whether this is due to the divergence of DNA binding and/or transcription activation, or the line did not express AtMS1 properly, or they may not be orthologous. Expression vaildation, DNA binding affinity tests, and domain swap experiments can be performed to clarify this.

3. MpGLID and Mp3g06700 are also expressed in the sporophyte. The epxression patterns of these genes during sporophyte development should be reported and discussed. Ideally, the characterization of the mutant phenotype in the sporophyte would be recommended but not required. Achieving expression of MpGLID specifically only in antheridia and not in the sporophyte can be possibly accomplished by utilizing the antheridia-specific promoter (e.g., MpBNB promoter) or conditional knockdown.

4. It is essential to examine charophyte algae to claim the role in gamete specification as ancestral. However, the authors can simply tone down their claim in the manuscript without providing new data.

5. There are several key citations regarding BNB and GLID missing in the manuscript. Please carefully review them and cite them accordingly.

Please contact us if there are ambiguous comments or if you wish to discuss the revision.

Given the nature of the comments, we are offering you 60 days from when we have issued this decision to complete the revision. If a revision is not returned within this time frame, and if you have not been granted an extension, we will withdraw the manuscript, which will leave you free to submit the work elsewhere. We are aware that it likely takes more than 60 days to obtain additional experimental data. If you need an extension, we encourage you to contact us at any point before the 60 days have passed.

When you are ready to submit the revised version, please upload a highlighted copy that indicates all changes made in response to the editor and reviewer recommendations. Include an itemized list of all changes made in response to each of the reviewer's suggestions in the "Response to Reviewers" section; please note that reviewers do not have access to your cover letter, nor was this decision letter shared with them.

Thank you for the privilege of reviewing your work. We look forward to receiving your revised manuscript.

Sincerely,

The Plant Cell Board of Editors

Please note the following:

-The Plant Cell now requires authors to complete and submit an author revisions checklist upon submission of a revised manuscript. The aim of the checklist is to aid authors in preparing a high-quality manuscript, facilitate the review and assessment of revised manuscripts, and help to ensure that journal standards are maintained across the board. If your manuscript is accepted, the completed checklist will be published as supplemental material attached to the article online. Please download a copy of the checklist (pdf fillable form) at this link, for submission with your revised manuscript: https://tpc.msubmit.net/html/Author_Revisions_Checklist.pdf.

-Supplemental materials should be restricted to large datasets and tables, presentation of replicates, and validation of reagents, methods, or genotypes. Any data that are used to support the major claims must be in the main manuscript. Supplemental figure legends must indicate what figure in the main manuscript is supported by the supplemental data presented. Please justify how each of the supplemental figures meet the criteria.

-Sampling methods and nature of "biological replicates" should be described precisely (i.e. different plants, parts of plants, pooled tissue, independent pools of tissue, sampled at different times, etc), along with a clear description of and rationale for any statistical analyses conducted. The reader should know exactly what was sampled; what forms the basis of the calculation of any means and statistical parameters reported. This is also necessary to ensure that proper statistical analysis was conducted.

-Want to add this revision deadline to your calendar? Click below!

-- Reviewer comments:

Reviewer #1 (Comments for the Author):

The study by Ren et al. discovered a key regulator of germ cell specification in land plants. They picked up a PHD-finger type transcription factor, MpGLID, from its preferentially expression in sperm cell precursors of the liverwort Marchantia polymorpha. Genetic analysis clearly indicated that MpGLID is required for germ cell differentiation and sufficient for inducing ectopic germ-cell like cells in the vegetative tissue. Further, the authors raised multiple evidence which indicates that MpBONOBO, a master regulator for germline fate initiation, directly activate MpGLID expression. AtMS1, a MpGLID homolog in Arabidopsis thaliana, failed to rescue Mpglid mutants, implicating functional divergence between these homologous genes.
The work significantly advanced our knowledge on mechanisms regulating germ cell specification in land plants, which is strongly supported by lines of evidence, however, one of the major claims "rewiring of genetic module" during land plant evolution is not supported well by the results shown in the current manuscript. It would be great if the authors could address the points below to support their claim.

Major points

1. Page 11, lines 321-326, Suplemental figure 11: it is difficult to conclude that AtMS1 is unable to rescue Mpglid mutants without showing that the cross-complementation experiment went well. Expression of AtMS1 in the transgenic plants should be confirmed at least by RT-PCR for mRNA level, and if possible, by western blot for protein level. I understand that it is difficult to detect AtMS1 protein in the transgenic plants since AtMS1 is expressed without tags. I wonder if the authors also tried to express tagged version of AtMS1 in Mpglid mutants. The other way to prove functionality of MpGLID promoter is to complement Mpglid mutants by the proMpGLID:MpGLID construct. Did the author tried it or is there any technical difficulty to do this experiment?

2. Related to the point 1 above, if AtMS1 really cannot rescue the Mpglid mutant, then what makes difference between AtMS1 and MpGLID? Would it be possible to extract any information explaining potential functional difference between them from amino acid sequence alignment of PHD-finger proteins in the tree in the supplemental figure 11A? e.g. some amino acid residues are only conserved among bryophytes or vascular plants. Are there any studies on how PHD-finger type proteins bind to DNA or chromatin?

3. page 12, lines 371-372: "BNB-GLID module predates the ancestor of land plants" I agree with conservation of BNB-GLID module among bryophytes, and hence the module would predate the divergence of different bryophytes, however, if the module predate the divergence of bryophytes and vascular plants, the same module would be retained in extant green algae as well. Do the authors have any evidence for it? Also, discussion here will become stronger if the authors mention a recent paper describing PpBNB function, which supports conserved function of BNB homologs in germline specification (PMID: 34942024).

4.Page 13, lines 378-384: Here the authors discuss a possible scenario on how BNB-GLID module functuin evolved

during land plants diversification. Another scenario would be that ancestral GLID/MS1 regulates germ line entry as well as spore formation, and in vascular plants MS1 function was restricted to microspore formation, while GLID function in bryophytes is restricted to germline initiation or GLID function is retained as ancestral but spacio-temporally separated to germline formation in the gametophytes and spore formation in sporophytes. The previous study on PpMS1a/b discovered that MS1a/b are expressed not only in gametophytes but also expressed in the sporogenous cells in the sporophyte. Although we do not know whether MS1a/b function in spore formation due to mutants' lethality, they might have function in spore formation in extant bryophytes. Interestingly, public transcriptome data for M. polymorpha indicate that MpGLID is expressed in the sporophyte. Therfore, it would be great if detailed expression pattern of MpGLID in the sporophyte is examined by using MpGLID promoter reporter.

From points 1-4, I raised issues related to evolutional scenario on how BNB-GLID module evolved. As I mentioned, experimental results found in the current manuscript is not enough to support the claim that ancestral GLID function was to regulate germline formation and it was co-opted to promote sporogenesis in flowering plants. If the authors have difficulty to provide further evidence support their claim, then the claim should be toned-down or at least the other possible scenario should be discussed equally. The title of the manuscript can be used as it is, but I prefer title telling the important discovery that the BNB-GLID module regulates germline formation.

5. page 8, lines 241-247: Figure 5D, how did the author generate the line (proEF1:BNB in glid mutants)? Please describe the details of this experiments in the corresponding part in the method section. If the BNB overexpressing construct was introduced to one of the Mpglid allele, evidence of BNB overexpression at least the level of mRNA should be provided. Or did the authors observe other BNB overexpression phenotype in the line, such as gamentangiophore formation in non-inductive condition?

6. Figure5H. Since it is reported that MpBNB acts in a heterodimer with MpLRL, I was wondering whether introduction of MpLRL in addition to MpBNB enhance transcription from the GLID promoter.

7. Figure3 G-J, Would it be possible to dissect out these ectopic antheridia-like structures, and perform RT-PCR analyses to detect several marker genes of antheridia?

Minor points

8. It would be better to swap the order of the Figure4 and Figure 5

9. page 5, lines 135-142, page 11, lines 334-342: it would be great if the authors refer to a paper by Higo et al. (PMID: 26858289). In the paper, the authors have performed comparative transcriptome analyses to identify genes expressed during antheridia development. They have identified MpGLID as a gene expressed in early antheridia and have performed in situ hybridization experiments to confirm results of transcriptome.

Reviewer #2 (Comments for the Author):

Rewiring of a genetic module regulating germline fate determination during land plant evolution
Ren et al

The authors investigate the function of MpGLID, encoding a plant homeodomain protein, in Marchantia by analysing the phenotype of loss- and gain-of-function alleles. MpGLID is shown to be required for proper antheridium and archegonium development and specification of both types of gametes. Ectopic expression in male vegetative thalli induces ectopic antheridium-like development in the normally vegetative thallus. Both male and female sterility precluded analysis of the sporophyte generation, where the gene is also highly expressed.

Specific comments
lines 92-3: it is not really the 'growth notches', but rather the apical meristem
line 101: differentiate rather than 'transform'
line 109: check this sentence for grammar
line 206-218: Higo et al (2016 Plant Cell & Physiology 57) seems to be missing as a reference - this study performed a similar analysis as the one presented in this paper, establishing benchmarks for stages of antheridial development as well as identifying a large number of genes expressed specifically in the antheridia. The present study should be compared to this earlier study - indeed the gene the authors call MpGLID was identified in Higo et al as one of 14 antheridium enriched transcription factors and Higo et al also note the paralog, Mp1g66170, is also antheridium enriched in its expression.
line 222: 'recent' is always relative
line 307: from the tree, the duplication appears to occur AFTER the split from chlorophyte, but before the streptophytes diversified

line 311: 'cluster' is not a valid phylogenetic term, clade might be better
lines 346-7: It might be mentioned that egg cells specification may be mediated by a RWP TF, e.g. see Koi et al 2016; Rövecamp et al 2016 - both in Current Biology.
lines 378-384: it is not clear how one can state that the gene was co-opted to tapetum development in angiosperms without knowing whether it performs the same function in liverworts/mosses. Also, in monophyly of bryophytes is assumed, the role in gamete specification could have been acquired in that lineage rather than being ancestral - would need to examine an outgroup in the charophycean algae to be sure.
There is another Arabidopsis gene, At1g33420, likely paralogous with MS1, which is more broadly expressed (according to TAIR) - if subfunctionalisation occurred, then this could retain some ancestral functions as well?

Reviewer #3 (Comments for the Author):

MS1 and MMD1 are two PHD-finger proteins essential for anther development and pollen formation in Arabidopsis. Previous investigation showed that their two homologues in the moss Physcomitrium patens, PpMS1A and PpMS1B, are together essential for male and female gametogenesis. In this work, Ren et al., identified a PHD-finger protein gene (GLID) from specifically expressed at early stage of antheridium development in in the liverwort Marchantia polymorpha. Through loss-of-function, overexpression and regulation analysis, they demonstrated that MpGLID under direct regulation of a bHLH TF gene BONOBO (MpBNB), a key switch to initiate the development of sexual reproductive branches, antheridiophores and archegoniophores, is a key regulator of germline specification in the liverwort. They further propose a model of BNB-GLID module in land plants evolution. This work provides novel insights of genes essential for plant sexual reproduction and evolution.

1. Since this manuscript focus on germline fate determination in plant, it will be helpful to provide recent progress of this field including MpBNB information in Introduction.

2. Moss Physcomitrium patens contain two PHD-finger protein genes PpMS1A and PpMS1B, which play redundant roles for male and female gametogenesis. Liverwort Marchantia polymorpha contains only one copy of this family gene?

3. Based on previous work, PpMS1A and PpMS1B are two homologues in the moss Physcomitrium patens of Arabidopsis MS1 and MMD1. This work did not provide any information that MpGLID is the ortholog of MS1 instead of MMD1. Why MpGLID is considered as homolog of AtMS1 in the working model in Figure 6.

4. AtMS1 is a very famous gene for anther development and pollen formation in Arabidopsis. In line 318-319, three publications between 2002-2007 were cited. Actually, much progress have been made recently. Please read recent publication about MS1 function for tapetum development and pollen formation.

5. In Arabidopsis, MS1 is under direct regulation of MYB80. However, MpGLID is under direct regulation of a bHLH TF MpBNB. It will be helpful to discuss or compare AtMS1/MpGLID based gene regulation network.

Reviewer #1 (Comments for the Author):

The study by Ren et al. discovered a key regulator of germ cell specification in land plants. They picked up a PHD-finger type transcription factor, MpGLID, from its preferentially expression in sperm cell precursors of the liverwort Marchantia polymorpha. Genetic analysis clearly indicated that MpGLID is required for germ cell differentiation and sufficient for inducing ectopic germ-cell like cells in the vegetative tissue. Further, the authors raised multiple evidence which indicates that MpBONOBO, a master regulator for germline fate initiation, directly activate MpGLID expression. AtMS1, a MpGLID homolog in Arabidopsis thaliana, failed to rescue Mpglid mutants, implicating functional divergence between these homologous genes.

The work significantly advanced our knowledge on mechanisms regulating germ cell specification in land plants, which is strongly supported by lines of evidence, however, one of the major claims "rewiring of genetic module" during land plant evolution is not supported well by the results shown in the current manuscript. It would be great if the authors could address the points below to support their claim.

Response: Thanks a lot for your positive assessment of our work. We have made revisions in response to your comments.

Major points

1. Page 11, lines 321-326, Suplemental figure 11: it is difficult to conclude that AtMS1 is unable to rescue Mpglid mutants without showing that the cross-complementation experiment went well. Expression of AtMS1 in the transgenic plants should be confirmed at least by RT-PCR for mRNA level, and if possible, by western blot for protein level. I understand that it is difficult to detect AtMS1 protein in the transgenic plants since AtMS1 is expressed without tags. I wonder if the authors also tried to express tagged version of AtMS1 in Mpglid mutants. The other way to prove functionality of MpGLID promoter is to complement Mpglid mutants by the proMpGLID:MpGLID construct. Did the author tried it or is there any technical difficulty to do this experiment?

Response: Thank you for raising this point. We have complemented Mpglid mutants with a proMpGLID:MpGLID construct and the results are included in Supplementary Figure 13 in the revised manuscript. Considering constitutive activity of CRISPR/Cas9 system, we performed complementation of Mp*glid*^{ge} mutants by expressing CRISPR/Cas9-resistant version of Mp*GLID* coding region (Mp*GLID*^{re}) driven by Mp*GLID* promoter (*pro*Mp*GLID:*Mp*GLID*^{re}). The defective phenotype of male and female gametogenesis was rescued, suggesting that the promoter of MpGLID gene (*pro*Mp*GLID*) properly functions in the transgenic plants investigated in our study.

2. Related to the point 1 above, if AtMS1 really cannot rescue the Mpglid mutant, then what makes difference between AtMS1 and MpGLID? Would it be possible to extract any information explaining potential functional difference between them from amino acid sequence alignment of PHD-finger proteins in the tree in the supplemental figure 11A? e.g. some amino acid residues are only conserved among bryophytes or vascular plants. Are there any studies on how PHD-finger type proteins bind to DNA or chromatin?

Responses: PHD-finger proteins represent one of the largest families of epigenomic effectors. This family is ubiquitous in eukaryotic organisms, and PHD-finger domains can recognize and bind specifically to histone modifications, activating or repressing target gene expression. (PMID: 21813457, 26156103). The functions of PHD proteins are versatile and involved in growth, development, and responses to stresses. A typical PHD-finger domain is characterized as an evolutionarily conserved cysteine-rich domain following an arrangement Cys4-His-Cys3 (shown in Supplementary Figure 14), forming a Zn^{2+}-binding domain, which can interact with modified histone tails (PMID: 37061424). Based on recognition of specific modifications, PHD fingers were divided into two groups: one recognizing H3K4me3, and the other one binding to unmodified H3 (PMID: 37061424).

The PHD-finger domain is the only recognized domain in PHD group B. To investigate the difference between AtMS1 and MpGLID, multiple sequence alignment has been performed using PHD domain amino acid sequences of group B proteins. Except for well conserved Cys4-His-Cys3 structure, it is difficult to identify the

difference between bryophytes and vascular plants, which possibly could be responsible for the potential functional difference between AtMS1 and MpGLID (Supplementary Figure 14).

Although AtMS1 and its orthologues in flowering plants have conserved roles in tapetal cell development and biosynthesis of pollen wall components, specific histone modification patterns or the target genes these proteins recognize, or directly regulate, are still unknown. Given that overexpression of Mp*GLID* induced dramatic changes of thalli in transgenic plants, to gain insight into functional difference between AtMS1 and MpGLID, PHD domain-swapping analysis was performed, and the chimeric proteins were ectopically expressed in *M. polymorpha* (Supplementary Figure 15). We found that the overexpression of chimeric proteins failed to phenocopy the transgenic plants overexpressing MpGLID proteins (Supplementary Figure 15, B). These observations suggest that functional differences between these two proteins may possibly be due to discrete changes in both N-terminals and PHD-finger domains. These results have been described (line 340-354) and new data included in Supplementary Figure 15 in the revised manuscript.

3. page 12, lines 371-372: "BNB-GLID module predates the ancestor of land plants" I agree with conservation of BNB-GLID module among bryophytes, and hence the module would predate the divergence of different bryophytes, however, if the module predate the divergence of bryophytes and vascular plants, the same module would be retained in extant green algae as well. Do the authors have any evidence for it? Also, discussion here will become stronger if the authors mention a recent paper describing PpBNB function, which supports conserved function of BNB homologs in germline specification (PMID: 34942024).

Responses: Thank you very much for your comments to improve our manuscript. We apologies for our ambiguous description as our purpose is to communicate the evolution of BNB-GLID in land plants. Functional analysis of PpBNB (PMID: 34942024) and MpBNB (this study) indicate conserved function of BNB homologs in germline specification in land plants, suggesting that the function of BNB-GLID might be present in the last common ancestral land plant shared by bryophytes and vascular plants. The last common ancestors were complex terrestrial plants (PMID: 36175544). The revised text reads “BNB proteins are members of bHLH subfamlily

VIIIa, which is land-plant specific (Pires and Dolan, 2010; Bowman et al., 2017; Yamaoka et al., 2018). There are two BNB proteins in *P. patens*, and loss of PpBNB functions led to interruption of gametogenesis (Sanchez-Vera et al., 2022), suggesting the conserved function of BNB proteins in germline specification in land plants. Therefore, it is suggested that the function of the BNB-GLID module in determining male germline fate in the haploid gametophyte generation may be present in the last ancestral land plant from which bryophytes and vascular plants were derived, and has been retained in bryophytes (Figure 6)". We hope that this revised text more clearly communicates the intended meaning.

4.Page 13, lines 378-384: Here the authors discuss a possible scenario on how BNB-GLID module functuin evolved during land plants diversification. Another scenario would be that ancestral GLID/MS1 regulates germ line entry as well as spore formation, and in vascular plants MS1 function was restricted to microspore formation, while GLID function in bryophytes is restricted to germline initiation or GLID function is retained as ancestral but spacio-temporally separated to germline formation in the gametophytes and spore formation in sporophytes. The previous study on PpMS1a/b discovered that MS1a/b are expressed not only in gametophytes but also expressed in the sporogenous cells in the sporophyte. Although we do not know whether MS1a/b function in spore formation due to mutants' lethality, they might have function in spore formation in extant bryophytes. Interestingly, public transcriptome data for M. polymorpha indicate that MpGLID is expressed in the sporophyte. Therfore, it would be great if detailed expression pattern of MpGLID in the sporophyte is examined by using MpGLID promoter reporter.

Responses: Thank you so much for your insightful comments. We adopted your helpful suggestion, and revised the text to make the likely scenario clearer in the Discussion of the revised manuscript We induced sporophyte formation by crossing between male and female MpGLID promoter reporter lines. The detailed expression pattern of Mp*GLID* during sporophyte development was carefully examined. The MpGLID promoter was active during sporophyte development, particularly in the early developing embryo, the foot cells of young sporophyte, and in the developing sporangium. Images are included as Supplemental Figure 18. These observations suggest that MpGLID and its orthologues in bryophytes may potentially play roles in

sporophyte development and/or sporogenesis in bryophytes, in addition to germline cell specification in the haploid gametophyte generation.

From points 1-4, I raised issues related to evolutional scenario on how BNB-GLID module evolved. As I mentioned, experimental results found in the current manuscript is not enough to support the claim that ancestral GLID function was to regulate germline formation and it was co-opted to promote sporogenesis in flowering plants. If the authors have difficulty to provide further evidence support their claim, then the claim should be toned-down or at least the other possible scenario should be discussed equally. The title of the manuscript can be used as it is, but I prefer title telling the important discovery that the BNB-GLID module regulates germline formation.

Responses: Thank you very much for your suggestions to improve our manuscript. According to our responses above, our title claim has been toned-down, and the likely scenario has also been discussed in our revised manuscript. In the new version of our manuscript, we have adopted your suggestion and changed the title to "The BNB-GLID module regulates germline formation in the liverwort *Marchantia polymorpha*", highlighting the main discovery of our study.

5. page 8, lines 241-247: Figure 5D, how did the author generate the line (proEF1:BNB in glid mutants)? Please describe the details of this experiments in the corresponding part in the method section. If the BNB overexpressing construct was introduced to one of the Mpglid allele, evidence of BNB overexpression at least the level of mRNA should be provided. Or did the authors observe other BNB overexpression phenotype in the line, such as gamentangiophore formation in non-inductive condition?

Response: We apologize for inadequate description of transgenic plant generation in submitted manuscript. We transformed *M. polymorpha* sporelings with three plasmids at the same time, including two plasmids (pB45-proMpEF1α-hCas9 and pB34-gRNA) for Mp*GLID* interruption by CRISPR/Cas9 system and one plasmid (pB34-PmeI-proMpEF1α) for Mp*BNB* overexpression, and the transgenic plants overexpressing Mp*BNB* with Mp*GLID* interrupted were selected for further analysis by following approaches. Mp*GLID* interruption was confirmed by DNA sequencing, and

overexpression of Mp*BNB* was validated by qRT-PCR. Information on how to generate the transgenic plants has been included in Methods, and results are presented in Supplementary Figure 10 in the revised manuscript.

6. Figure5H. Since it is reported that MpBNB acts in a heterodimer with MpLRL, I was wondering whether introduction of MpLRL in addition to MpBNB enhance transcription from the GLID promoter.

Response: Thank you very much for your comments. We made several attempts to introduce MpLRL and MpBNB together with a reporter gene into tobacco leaves to test whether the transcription from the Mp*GLID* promoter could be enhanced, but they were not successful. However, failure of these experiments does not weaken our conclusion that MpBNB regulates germline cell specification by activating Mp*GLID* via directly binding to its promoter. Certainly , the question you raised is intriguing, but due to the limited time frame, we will revisit this issue in future.

7. Figure3 G-J, Would it be possible to dissect out these ectopic antheridia-like structures, and perform RT-PCR analyses to detect several marker genes of antheridia?

Response: Thank you for your suggestion. We tried to isolate these cells from somatic tissues of thalli. Unfortunately, we failed to achieve this because thalli overexpressing Mp*GLID* were too soft to manipulate. In addition, these cells are nearly indistinguishable from neighboring somatic cells in dissected tissues examined by light microscopy. Alternatively, we harvested thalli overexpressing Mp*GLID*, and checked expression of three representative genes by qRT-PCR, which was in line with our RNA-seq analysis. The result is included in the revised manuscript as Supplementary Figure 8.

Minor points

8. It would be better to swap the order of the Figure 4 and Figure 5

Response: Apologies for the mistake. The order of Figure 4 and Figure 5 have been

rearranged in our revised manuscript.

9. page 5, lines 135-142, page 11, lines 334-342: it would be great if the authors refer to a paper by Higo et al. (PMID: 26858289). In the paper, the authors have performed comparative transcriptome analyses to identify genes expressed during antheridia development. They have identified MpGLID as a gene expressed in early antheridia and have performed in situ hybridization experiments to confirm results of transcriptome.

Response: This has been included in the revised manuscript. Thank you very much for your suggestion to improve our manuscript.

Reviewer #2 (Comments for the Author):

Rewiring of a genetic module regulating germline fate determination during land plant evolution
Ren et al

The authors investigate the function of MpGLID, encoding a plant homeodomain protein, in Marchantia by analysing the phenotype of loss- and gain-of-function alleles. MpGLID is shown to be required for proper antheridium and archegonium development and specification of both types of gametes. Ectopic expression in male vegetative thalli induces ectopic antheridium-like development in the normally vegetative thallus. Both male and female sterility precluded analysis of the sporophyte generation, where the gene is also highly expressed.

Response: Thank you for your comments to improve our manuscript.

Specific comments
lines 92-3: it is not really the 'growth notches', but rather the apical meristem

Response: This has been corrected. Thank you for your suggestion.

line 101: differentiate rather than 'transform'

Response: Corrected.

line 109: check this sentence for grammar

Response: Apologies for this mistake. This has been corrected, and the revised text reads "The SCs continue proliferating in a nearly synchronous manner before they differentiate into spermatid mother cells (SMCs)".

line 206-218: Higo et al (2016 Plant Cell & Physiology 57) seems to be missing as a reference - this study performed a similar analysis as the one presented in this paper, establishing benchmarks for stages of antheridial development as well as identifying a large number of genes expressed specifically in the antheridia. The present study should be compared to this earlier study - indeed the gene the authors call MpGLID was identified in Higo et al as one of 14 antheridium enriched transcription factors and Higo et al also note the paralog, Mp1g66170, is also antheridium enriched in its expression.

Responses: Apologies for missing this important reference. We are assuming that the gene you referred is the *Marchantia polymorpha* PHD gene (Mp3g06700) belonging to subclade II. The reference has been introduced, and the text revised (line 380-388) to give the reason why Mp3g06700 did not show up in our analysis. The text reads "The functions of genes from subclade II of PHD family clade IIa have not been reported to date. Mp3g06700 from this subclade was also identified as antheridium-specific transcription factor by Higo et al. (2016). However, this gene was not identified from the criteria (specific expression in antheridium and maximal abundance at early stage antheridium development), we used to search for candidate genes most likely involved in specification of spermatogenous cells. The transcripts of Mp3g06700 gradually accumulated during antheridium development and reached maximum abundance only at late stage, which was 4-fold greater than in early-stage antheridia (Supplemental Figure 16, A)".

line 222: 'recent' is always relative

Response: Removed.

line 307: from the tree, the duplication appears to occur AFTER the split from chlorophyte, but before the streptophytes diversified

Response: Apologies for this mistake. It has been corrected, and the revised text reads "MpGLID belongs to the ancient PHD family clade IIa (Cao et al., 2018; Landberg et al., 2022), which has split into two subclades (I and II) after divergence of streptophyte lineages from chlorophyte algae, but before the stretophytes diversified (Supplemental Figure 13, A)".

line 311: 'cluster' is not a valid phylogenetic term, clade might be better

Response: Thank you for pointing this out. The revised term "clade" is used.

lines 346-7: It might be mentioned that egg cells specification may be mediated by a RWP TF, e.g. see Koi et al 2016; Rövecamp et al 2016 - both in Current Biology.

Response: Thank you for your suggestion to improve our manuscript. The information has been introduced, and the revised text reads "A group of RWP-PR domain (RKD) transcription factors were implicated to mediate egg cell specification, and ectopic overexpression of some *RKD* genes in Arabidopsis was able to induce cell proliferation with an egg cell-specific transcription profile (Kőszegi et al., 2011; Koi et al., 2016; Rövekamp et al., 2016)"

lines 378-384: it is not clear how one can state that the gene was co-opted to tapetum development in angiosperms without knowing whether it performs the same function in liverworts/mosses. Also, in monophyly of bryophytes is assumed, the role in gamete specification could have been acquired in that lineage rather than being ancestral - would need to examine an outgroup in the charophycean algae to be sure. There is another Arabidopsis gene, At1g33420, likely paralogous with MS1, which is more broadly expressed (according to TAIR) - if subfunctionalisation occurred, then

this could retain some ancestral functions as well?

Responses: Thank you very much for your insightful comments, and apologies for not making this point clearly in submitted manuscript.

As Mp3g06700, orthologous to At1g33420, was specifically expressed in antheridium we generated Mp3g06700 mutants using the CRISPR/Cas9 approach. We found that male gametogenesis was not affected by the loss of Mp3g06700 function (Supplemental Figure 16), indicating that this gene does not contribute to male gametogenesis. As you mentioned, the Arabidopsis orthologue At1g33420 is almost ubiquitously expressed in *A. thaliana*, with relatively higher transcript abundance in seeds and shoot apex (Supplemental Figure 17). Collectively, these observations suggest that subclade II proteins possibly may not play major roles in male gametogenesis in land plants.

Studies in *P. patens* (Landberg et al., 2022; Sanchez-Vera et al., 2022) and *M. polymorpha* (Yamaoka et al., 2018; this study) suggested the conserved function of BNB and PHD group B members in germline specification in bryophytes. With the evidence we described, we suggest that the BNB-GLID module in germline specification in haploid gametophyte generation might be present in the most recent ancestral land plants shared by bryophytes and vascular plants. The last common ancestors were complex terrestrial plants (Harris et al., 2022), and in the revised manuscript, we have amended and revised the text to make this point clearly in the Discussion.

We totally agree that the ancestral function cannot be determined without investigation of an outgroup in the charophcean algea. For sure, further studies of genes in charophyte alga would help us to better understand the evolution of the PHD family clade IIa, which we are excited to look at in future.

Reviewer #3 (Comments for the Author):

MS1 and MMD1 are two PHD-finger proteins essential for anther development and pollen formation in Arabidopsis. Previous investigation showed that their two homologues in the moss Physcomitrium patens, PpMS1A and PpMS1B, are together

essential for male and female gametogenesis. In this work, Ren et al., identified a PHD-finger protein gene (GLID) from specifically expressed at early stage of antheridium development in in the liverwort Marchantia polymorpha. Through loss-of-function, overexpression and regulation analysis, they demonstrated that MpGLID under direct regulation of a bHLH TF gene BONOBO (MpBNB), a key switch to initiate the development of sexual reproductive branches, antheridiophores and archegoniophores, is a key regulator of germline specification in the liverwort. They further propose a model of BNB-GLID module in land plants evolution. This work provides novel insights of genes essential for plant sexual reproduction and evolution.

Response: Thanks a lot for your positive assessment of our work. We have made revisions in response to your comments.

1. Since this manuscript focus on germline fate determination in plant, it will be helpful to provide recent progress of this field including MpBNB information in Introduction.

Response: Thank you for your suggestion to improve our manuscript. Information has been added in Introduction in revised manuscript.

2. Moss *Physcomitrium patens* contain two PHD-finger protein genes PpMS1A and PpMS1B, which play redundant roles for male and female gametogenesis. Liverwort Marchantia polymorpha contains only one copy of this family gene?

Response: Phylogenomic analyses revealed ancestral gene duplications in mosses (Gao et al., 2022 J Syst Evol), and the whole-genome duplication events have occurred in ancestors of *Physcomitrium patens* (Rensing et al., 2008 Science; Lang et al., 2018 Plant Journal), which have contributed to the multiple copies of genes in moss genomes. We searched the *Marchantia polymorpha* genome for homologues with different parameters, using AtMS1, PpMS1A, and PpMS1B as queries. Based on our constant phylogenetic analysis, it was strongly supported that Mp*GLID* is the only gene in *M. polymorpha* which is orthologous to *AtMS1, PpMS1A*, and *PpMS1B* (Supplemental Figure 13, A). Please also see response below.

3. Based on previous work, PpMS1A and PpMS1B are two homologues in the moss Physcomitrium patens of Arabidopsis MS1 and MMD1. This work did not provide any information that MpGLID is the ortholog of MS1 instead of MMD1. Why MpGLID is considered as homolog of AtMS1 in the working model in Figure 6.

Response: We apologise for the poor description. Using 116 sequences from 36 representative species across green plants, we investigated the evolutionary history of PHD family clade IIa by constructing phylogenetic trees employing different approaches (RAxML, MrBayes). The trees displayed constant topology with strong support, and a maximum likelihood tree was showed as a representative in our submitted manuscript (Supplemental Figure 13, A). In our analyses, the PHD family clade IIa contains two subclades, subclade I and subclade II. MpGLID and its orthologues belong to subclade I, in which land plants proteins are divided into two groups, A (containing MMD1 and AT2G01810) and B (MS1 and MpGLID). We found that the genes from group A are only present in angiosperms, indicating that the counterpart genes have been lost in non-seed plants and gymnosperms. In group B, MpGLID and genes from non-seed land plants and gymnosperms form a sister clade to the genes from angiosperms. Taken together, these observations indicate that MpGLID is orthologous to AtMS1, but not MMD1.

4. AtMS1 is a very famous gene for anther development and pollen formation in Arabidopsis. In line 318-319, three publications between 2002-2007 were cited. Actually, much progress have been made recently. Please read recent publication about MS1 function for tapetum development and pollen formation.

Response: Thank you for your suggestion to improve the citations. References have been selected and cited in the revised manuscript.

5. In Arabidopsis, MS1 is under direct regulation of MYB80. However, MpGLID is under direct regulation of a bHLH TF MpBNB. It will be helpful to discuss or compare AtMS1/MpGLID based gene regulation network.

Response: Thank you very much for the suggestion. As one of the largest transcription factor families, MYB proteins play essential roles in the regulation of

various biological processes in plants. An extensive analysis of MYB protein evolution in plants (Jiang and Rao, 2020 Plant Physiol) indicated that two paralogues, AtMYB80 and AtMYB35, belong to two subclades of MYB VIII-B clade respectively, which diverged before the diversification of extant seed plants. Notably, there are no AtMYB80/AtMYB35 orthologues present in *M. polymorpha*. Instead, three genes were identified in *P.patens* as orthologues of AtMYB80/AtMYB35. Unfortunately, the function of these three genes has not been reported to date. Further elucidation of the roles of these genes in *P. patens* becomes essential to understand the evolution of VIII-B member function, which, in turn, will provide insights into the evolutionary regulation of AtMS1/MpGLID in land plants. Thank you for raising this point, and it is a very intriguing question to investigate in future.

Dear Dr. Bo Xu:

We have received reviews of your manuscript entitled "The genetic module BNB-GLID regulates germline fate determination in the liverwort Marchantia polymorpha." On the basis of the advice received, the board of reviewing editors would like to accept your manuscript for publication in The Plant Cell. This acceptance is contingent on revision based on the comments of our reviewers. In particular, please consider the following:

1. As Reviewer #1 pointed out, please update the methods section.
2. As Reviewer #2 pointed out, the results from Physcomitrium by Landberg et al. (2022) need to be clearly mentioned and integrated.
3. Because the sporophytic function is not completely clear, please include this point in the discussion where evolutionary scenarios/models are described.

Please highlight all changes and include a detailed annotation of changes of the text, with line numbers, and noting your responses to the comments.

To submit your revised manuscript, click:

Link Not Available

If you have any questions about the revision submission procedures, please contact the Editorial Office Staff (tpc-submissions@aspb.org). If you cannot return the revised manuscript within 30 days, please let us know. Otherwise, we will assume that you have elected not to revise the manuscript and withdraw it.

Thank you very much for the privilege of reviewing this work. I look forward to receiving the next version.

Sincerely,

The Plant Cell Board of Editors

Reviewer #1 (Comments for the Author):

In the revised manuscript, the authors thoroughly and clearly answered to all my comments with some additional data. Since the authors have done additional analysis using some transgenic plants which are not described in the first version, the authors should update the corresponding part of the method section. Other than that, I do not have any specific comments. I think that the revised manuscript shows robust evidence which supports their discovery of the genetic module regulating germline fate determination in land plants.

Reviewer #2 (Comments for the Author):

Rewiring of a genetic module regulating germline fate determination during land plant evolution
Ren et al

The results in this paper confirm the previous results in Physcomitrium AND advance our understanding in that MpGLID is a target of MpBNB, providing insight into the transcriptional cascade leading to gamete production. In addition, it is shown that the MpGLID paralog does not appear to have a role in male gametogenesis. The former should be better integrated (see below), while the latter can be highlighted.
The sporophytic functions remain enigmatic - and thus, some of the evolutionary conclusions will have to wait until phenotypes can be observed in this generation.

specific comments:
lines 82-87: should also reference the Landberg 2022 paper on Physcomitrium, as it is essentially the same result as reported here in Marchantia. Lines 409-410 are a bit disingenuous given the similarity in results between the present paper and Landberg et al. This also applies to the discussion: in lines 386-424 the results of Marchantia and Physcomitrium should be better integrated.

Reviewer #3 (Comments for the Author):

Authors have performed the analysis suggested and answered my questions.

Please also note the following:

-The Plant Cell now requires authors to complete and submit an author revisions checklist upon submission of a revised manuscript. The aim of the checklist is to aid authors in preparing a high-quality manuscript, facilitate the review and assessment of revised manuscripts, and help to ensure that journal standards are maintained across the board. If your manuscript is accepted, the completed checklist will be published as supplemental material attached to the article online. Please download a copy of the checklist (pdf fillable form) at this link, for submission with your revised manuscript: https://tpc.msubmit.net/html/Author_Revisions_Checklist.pdf.

-Supplemental materials should be restricted to large datasets and tables, presentation of replicates, and validation of reagents, methods, or genotypes. Any data that are used to support the major claims must be in the main manuscript. Supplemental figure legends must indicate what figure in the main manuscript is supported by the supplemental data presented. Please justify how each of the supplemental figures meet the criteria.

-Sampling methods and the nature of "biological replicates" should be described precisely (i.e. different plants, parts of plants, pooled tissue, independent pools of tissue, sampled at different times, etc.), along with a clear description of and rationale for any statistical analyses conducted. The reader should know exactly what was sampled; what forms the basis of the calculation of any means and statistical parameters reported. This is also necessary to ensure that proper statistical analysis was conducted.

Reviewer #1 (Comments for the Author):

In the revised manuscript, the authors thoroughly and clearly answered to all my comments with some additional data. Since the authors have done additional analysis using some transgenic plants which are not described in the first version, the authors should update the corresponding part of the method section. Other than that, I do not have any specific comments. I think that the revised manuscript shows robust evidence which supports their discovery of the genetic module regulating germline fate determination in land plants.

Response: Thanks a lot for your positive assessment of our work. We are happy that you agree with our findings and interpretations. Information has been updated in the Methods section for additional analyses in the revised manuscript.

Reviewer #2 (Comments for the Author):

Rewiring of a genetic module regulating germline fate determination during land plant evolution
Ren et al

The results in this paper confirm the previous results in Physcomitrium AND advance our understanding in that MpGLID is a target of MpBNB, providing insight into the transcriptional cascade leading to gamete production. In addition, it is shown that the MpGLID paralog does not appear to have a role in male gametogenesis. The former should be better integrated (see below), while the latter can be highlighted. The sporophytic functions remain enigmatic - and thus, some of the evolutionary conclusions will have to wait until phenotypes can be observed in this generation.

Response: Thank you very much for your appreciation. It has been clearly mentioned that further elucidation of MpBNB and MpGLID functions in the sporophyte will be helpful to better understand the evolution of the BNB-GLID module in land plants.

specific comments:
lines 82-87: should also reference the Landberg 2022 paper on Physcomitrium, as it is

essentially the same result as reported here in Marchantia. Lines 409-410 are a bit disingenuous given the similarity in results between the present paper and Landberg et al. This also applies to the discussion: in lines 386-424 the results of Marchantia and Physcomitrium should be better integrated.

Response: Thank you very much for your comment to improve our manuscript. The results from *Physcomitrium patens* by Landberg et al. (2022) have been clearly described and integrated in our revised manuscript as suggested.

Reviewer #3 (Comments for the Author):

Authors have performed the analysis suggested and answered my questions.

Response: Thanks a lot for your positive assessment of our work. We are happy that you agree with our findings and interpretations.

Dear Dr. Bo Xu:

We are pleased to inform you that your paper entitled "The genetic module BNB-GLID regulates germline fate determination in the liverwort Marchantia polymorpha" has been accepted for publication in The Plant Cell, pending a final editorial review by a science editor. At this stage, your manuscript will be evaluated by a science editor with respect to its presentation of scientific content, compliance with journal policies, and presentation for a broad readership. The Plant Cell has contracted with Plant Editors (planteditors.com) to provide this service to our authors, and you will soon receive additional information on this process.

Please note that each author needs to link their ORCID identifier to their account in the system before your manuscript can be published. If any authors do not have an ORCID linked to their account, they will receive a message with a link to complete this task. Please ensure that ALL of your coauthors have completed this task as soon as possible.

ASPB offers an OPEN option that allows authors to have their online articles available for free to all users immediately upon publication. For more information about the ASPB OPEN option, refer to the Final Submission Checklist Form.

The Plant Cell and The Arabidopsis Information Resource (TAIR) are collaborating to collect functional annotation data about Arabidopsis genes from authors. This includes information about the gene's molecular function (e.g., kinase activity, ATP synthetase activity), the biological process/es it is involved in (e.g., endosperm development, threonine biosynthesis), its subcellular location (e.g., nucleus, ER), anatomical or developmental expression pattern (e.g., leaf, ovule, flower stage 10, seedling stage), or its partner in a protein-protein interaction (e.g., AT1G01010 interacts with AT1G01020).

If your paper contains results falling into one or more of these categories for Arabidopsis genes, we request that you now submit these data for inclusion in TAIR by filling in the form provided at the following URL: https://www.arabidopsis.org/doc/submit/functional_annotation/123. If you need further clarification on what types of data can be submitted please contact curator@arabidopsis.org.

Finally, we encourage your submission of artwork for the journal cover. Monthly issues will have an online cover image and selected covers will be used for posters, other promotional items, and "wallpaper" for mobile devices. For more information, refer to Cover Submission in the Instructions for Authors https://tpc.msubmit.net/cgi-bin/main.plex?form_type=display_auth_instructions.

We look forward to seeing your paper published.

Sincerely,

The Plant Cell Board of Editors

=====
IMPORTANT REMINDER: PEER REVIEW REPORTS
=====

If you opted to publish a peer review report along with your article during the original submission process, it will be prepared by the editorial staff and publicly posted with your manuscript, inside the zip file that contains any other supplemental material. As a reminder, the peer review report is a public record of all comments from editors and reviewers, as well as your prior responses, as you received them in the decision letters for each draft of your manuscript. If you agreed to publish this report and have changed your mind, or are not sure if you selected this option, please contact the editorial office as soon as possible before signing the license agreement from our publisher.

=====

----FOR ASPB OFFICE USE ONLY (DO NOT EDIT)----
MSID: 38209
Corresponding Author: Dr. Xu
